# Multiscale Invasion Assay for Probing Macrophage Response to Gram-Negative Bacteria

**DOI:** 10.3389/fchem.2022.842602

**Published:** 2022-02-15

**Authors:** Kimberly A. Wodzanowski, Jeffrey L. Caplan, April M. Kloxin, Catherine L. Grimes

**Affiliations:** ^1^ Department of Chemistry and Biochemistry, University of Delaware, Newark, DE, United States; ^2^ Department of Biological Sciences, University of Delaware, Newark, DE, United States; ^3^ Bioimaging Center, Delaware Biotechnology Institute, Newark, DE, United States; ^4^ Department of Chemical and Biomolecular Engineering, University of Delaware, Newark, DE, United States; ^5^ Department of Materials Science and Engineering, University of Delaware, Newark, DE, United States

**Keywords:** biomaterials, bioorthogonal chemistry, invasion model, bacteria-macrophage interactions, click chemistry, live cell imaging, 3D cell culture, synthetic extracellular matrix

## Abstract

The immune system is a complex network of various cellular components that must differentiate between pathogenic bacteria and the commensal bacteria of the human microbiome, where misrecognition is linked to inflammatory disorders. Fragments of bacterial cell wall peptidoglycan bind to pattern recognition receptors within macrophages, leading to immune activation. To study this complex process, a methodology to remodel and label the bacterial cell wall of two different species of bacteria was established using copper (I) catalyzed azide-alkyne cycloaddition (CuAAC) and strain-promoted azide-alkyne cycloaddition (SPAAC). Additionally, an approach for three-dimensional (3D) culture of human macrophages and their invasion with relevant bacteria in a well-defined hydrogel-based synthetic matrix inspired by the microenvironment of the gut was established. Workflows were developed for human monocyte encapsulation and differentiation into macrophages in 3D culture with high viability. Bacteria invaded into macrophages permitted *in situ* peptidoglycan labeling. Macrophages exhibited biologically-relevant cytokine release in response to bacteria. This molecularly engineered, multi-dimensional bacteria-macrophage co-culture system will prove useful in future studies to observe immunostimulatory, bacterial fragment production and localization in the cell at the carbohydrate level for insights into how the immune system properly senses bacteria.

## Introduction

**FIGURE 1:**
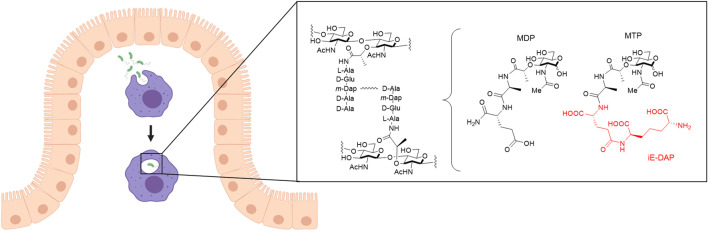
Invasion of bacteria into macrophages. Macrophages (purple) that arise from monocytes reside in the connective tissue underlying the intestinal epithelium (beige). Macrophages phagocytose pathogens such as bacteria (green) and break them down into smaller fragments to determine what type of signaling for an immune response is needed. One recognition element of bacteria, peptidoglycan (PG), is composed of alternating *N*-acetyl glucosamine (NAG) and *N*-acetyl muramic acid (NAM) sugars with a pentapeptide chain off the 3-position of the NAM that is further crosslinked to form mature PG. Out of this larger structure, smaller synthetic fragments, muramyl dipeptide (MDP) and muramyl tripeptide (MTP), can be teased out with the core NAM sugar, which are known to elicit an immune response. D-glutamyl-meso-diaminopimelic acid (iE-DAP), shown in red, is a specific muropeptide in Gram-negative bacteria that activates the NLR, NOD1, to initiate an immune response. Figure created with Biorender.

The innate immune system is the body’s first line of defense against invading pathogens. It is armed with sophisticated molecular mechanisms to sense and differentiate between pathogenic bacteria and the over 39 trillion bacteria constituting the human microbiome. Macrophages have many roles in the innate immune system, including ingesting pathogens by phagocytosis, scavenging dead cells and cell debris, and remodeling tissues after injury (Galli et al., 2011). Based on their tissue location and specialization in various microenvironments, macrophages take on a variety of names including alveolar macrophages (lung), microglia (brain and central nervous system), osteoclasts (bone), and Kupffer cells (liver) to name a few (Italiani and Boraschi, 2014). Macrophages are derived from monocytes, which circulate in the blood stream until entering the tissue and differentiating into macrophages, based on release of activating lymphokines from T lymphocytes present in the area of infection (Johnston, 1988). Mature macrophages express receptors that identify pathogens, allowing their proper uptake into the cell for degradation and response (Galli et al., 2011). These receptors include membrane-associated Toll-like receptors (TLRs) and cytosolic NOD-like receptors (NLRs), which are known to bind to fragments of the bacterial peptidoglycan (PG), a component of the bacterial cell wall. Peptidoglycan structure generally contains alternating sugars *N*-acetyl glucosamine (NAG) and *N*-acetyl muramic acid (NAM) with a pentapeptide chain off of the NAM sugar (Figure 1). Chemists have developed synthetic PG mimics of smaller fragments such as muramyl dipeptide (MDP) and muramyl tripeptide (MTP), which bind to NLRs and therefore have been used to study immune responses (Figure 1). Misrecognition of various PG fragments by the immune system is hypothesized to lead to diseases including Crohn’s disease, inflammatory bowel disease, asthma, and gastrointestinal cancers (Geddes et al., 2009; Thaiss et al., 2016). Although MDP and MTP serve as important tools in studying immune responses in humans, the true identity of naturally produced immunostimulatory fragments, how they are generated, and how they interact with innate immune receptors in macrophages is not well known (Hasegawa et al., 2006; Herskovits et al., 2007; Humann and Lenz, 2009). Recently, underlying inflammation has been suggested to have other fundamental roles in biology that have yet to be discovered (Medzhitov, 2021). Therefore, there is a need for development of physiologically-relevant co-culture systems to model invasion of bacteria within human macrophages and begin probing these complex interactions toward addressing these important questions. Moreover, these systems should be amenable to downstream chemical biology labeling techniques of both the host and invading bacterial species.

In a typical experiment to study macrophage-bacteria interactions, macrophages are cultured on two-dimensional (2D) surfaces such as tissue culture polystyrene (TCPS). While this approach provides a well-defined environment, the material is unnaturally polarizing and has mechanical properties (Young’s modulus (E) ∼ 3 GPa) a million times stiffer than those of the native soft tissue of the intestines (E ∼ 2–20 kPa) (Simon-Assmann et al., 1995; Rehmann and Kloxin, 2013). Seminal works have demonstrated how the function of many different cell types (e.g., stem cells, epithelial cells, cancer cells) is influenced by the dimensionality and stiffness of the culture environment (Petersen et al., 1992; Tanaka et al., 2004; Engler et al., 2006). Soft three-dimensional (3D) culture systems have been shown to be particularly effective for probing cell differentiation and migration in a physiologically relevant manner (Tibbitt and Anseth, 2009). As macrophages reside within connective tissue underneath the epithelium in the gut and respond to bacteria that breach this layer, 3D migration and interactions with the extracellular matrix (ECM) play an important role in infection clearing, suggesting the potential importance of studying macrophage response to bacterial invasion in multi-dimensional systems (Wodzanowski et al., 2020).

For studies of the gut in 3D, recent progress has been made in the development of organoids cultures, amongst other approaches (Snyder et al., 2020; Wodzanowski et al., 2020). Organoids are 3D cell clusters formed *in vitro*, with stem cells or cells derived from primary tissues, and are capable of self-organization and self-renewal, exhibiting similar function to *in vivo* organs (de Souza, 2018). However, organoids lack immune cells, in addition to requiring lengthy timescales with intensive maintenance to fully generate (e.g., 1–3 months) (Clevers, 2016; de Souza, 2018). Animal models have also provided insights in the context of inflammatory bowel disorders (IBD) by allowing study of mucosal inflammation. However, these models can have reproducibility issues and do not sufficiently capture human IBD as they cannot accurately control for intestinal pathology, inflammation, and bacteria related to IBD (Blumberg et al., 1999; Goyal et al., 2014; Webb, 2014; Wodzanowski et al., 2020). Currently, a gap remains in physiologically-relevant, multi-dimensional systems for studying specific bacteria-immune cell interactions, where few immune cells, particularly macrophages, have been cultured in three dimensions (Sun et al., 2011; Blasioli et al., 2014; Peck et al., 2014; Samavedi et al., 2017). There is a need for robust hybrid systems with well-defined 3D properties that can be manipulated to reflect aspects of the native tissue and are suitable for co-culture of macrophages and bacteria.

Hydrogels, crosslinked water-swollen networks of hydrophilic polymers, have emerged as good candidates for mimicking a variety of soft tissue microenvironments for 3D cell culture applications. In particular, synthetic hydrogels can be engineered to mimic key properties of the native ECM, including mechanical properties and biochemical content, and permit 3D cell encapsulation and culture (Caliari and Burdick, 2016). These systems include multi-arm polymers (e.g., biologically-inert poly (ethylene glycol) (PEG)) that have been functionalized with reactive handles (e.g., (meth)acrylates, norbornenes, vinyl sulfones, thiols) for crosslinking with functionalized cell-degradable and integrin-binding peptides inspired by native tissues for a range of applications (Caliari and Burdick, 2016). Studies of macrophages with such synthetic materials often have focused on probing the foreign body response in 2D culture studies (Blakney et al., 2012; Witherel et al., 2019), given its importance in the design of implanted materials, with recent efforts demonstrating feasibility of macrophage 3D culture (Samavedi et al., 2017; Kim et al., 2019). More recently, matrix-metalloproteinase (MMP) degradable synthetic hydrogels have been utilized to encapsulate human epithelial-mesenchymal intestinal organoids for coculture studies (Jowett et al., 2021). Additionally, 3D cocultures are beginning to be developed to study bacterial infections such as the case of the oral microbiome and the lung (Montefusco-Pereira et al., 2020; Mountcastle et al., 2020). These versatile materials provide significant opportunities for creating multi-dimensional culture systems with well-defined and tunable properties for probing macrophage-bacteria interactions to test hypotheses in more physiologically-relevant environments related to the gut.

In parallel, novel methods have emerged to study the bacterial cell wall. Work has been done previously to label various aspects of PG including small fluorophores coupled to D-amino acids, NAG and NAM sugar probes, and near-infrared radiation (NIR) fluorogenic probes (Wodzanowski et al., 2020; Banahene et al., 2021). Because synthetic fragments MDP and MTP both contain the NAM sugar, it is believed that NAM plays an important structural role in fragment identification and subsequent infection clearing by macrophages. NAM probes developed have had a variety of functional groups including bioorthogonal handles, fluorescent dyes, biotin, and photoactivatable crosslinker (DeMeester et al., 2018). Of particular interest are the click handles, especially the bioorthogonal azide at the 2-acetyl position, providing versatility in what is ‘clicked’ onto the handle for various assays (Liang et al., 2017; DeMeester et al., 2019). Another advantage of the NAM probes is that under a lethal dose of the antibiotic fosfomycin, the first enzyme in the PG biosynthetic pathway, Mur A, can be selectively inhibited, which prevents first committed PG biosynthetic pathway steps from occurring, and cells only survive if they uptake supplemented NAM sugar provided through alternative recycling machinery if present in the cell (Kahan et al., 1974). Instrumental work by Mayer and coworkers showed that certain *Pseudomonas* species contain recycling machinery involving the enzymes anomeric NAM/NAG kinase (AmgK) and α-1-phosphate uridylyl transferase (MurU) that under a lethal dose of Fosfomycin can recycle NAM into uridine diphosphate (UDP)-NAM, which is then incorporated into mature PG (Gisin et al., 2013). Therefore, utilizing these NAM probes can allow visualization of the bacterial PG core and have potential downstream applications as an affinity handle for purifying naturally produced PG fragments from breakdown in macrophages.

In this work, we aimed to establish a well-defined, bio-inspired co-culture system that enabled encapsulation and culture of immune cells relevant to the human gut, and to study their response to invasion by Gram-negative bacteria in three dimensions. First, we utilized the biorthogonal azide NAM probe to remodel and label bacterial PG, including for the first time the pathogen *P. aeruginosa*, through copper (I) azide alkyne cycloaddition (CuAAC) and strain-promoted azide-alkyne cycloaddition (SPAAC) click reactions. Next, we developed a PEG-peptide hydrogel composition inspired by aspects of the microenvironment of the healthy gut. We established workflows for the successful encapsulation of human monocytes and their differentiation into human macrophages within these hydrogel-based synthetic matrices where cells can be facilely recovered by digestion of the hydrogel with collagenase for downstream assays. Utilizing the fluorescent bacteria, we visualized macrophage engulfment of bacteria in real time and in both 2D and 3D culture. Further, we probed macrophage cytokine expression in these systems with contrasting dimensionality, elucidating key differences in macrophage response. These studies provide new tools, such as an expansion of previous fluorescent labeling strategies of bacteria and a new 3D model system, for insights into the interactions between bacteria and immune cells and how our immune system recognizes bacteria in a variety of physiologically relevant settings.

## Materials and Methods

See supplemental information (SI) for general materials and methods.

### Bacterial Labeling and Remodeling With SPAAC

Overnight pre-cultured *E. coli ΔMurQ-KU* cells (Liang et al., 2017) or *P. aeruginosa* were inoculated into fresh LB medium and were incubated until the OD_600nm_ was about 0.600. 1.2 ml of cell suspension were collected by centrifugation at 8,000 rpm (6,000 g) for 5 min. *E. coli ΔMurQ-KU* cells were resuspended in 190 μl LB medium. 6 mM NAM sugar (NAM (Sigma-Aldrich) or AzNAM (synthesized in house based on established protocols) (Liang et al., 2017)), 1 mM isopropyl-1-thio-β-d-galactoside (*E. coli* only) and 200 μg/ml fosfomycin were added into cell samples. Cells were incubated while shaking at 37 °C for 60 min. Cells were then collected (10,000 rpm, 2 min) and washed with 2 × 600 μl 1xPBS. Cells were resuspended in 194 μl LB and then 30 μM DBCO-488 (AZDye™ 488 DBCO; Click Chemistry Tools) was added. Cells were covered and incubated at 37°C for 40 min while shaking. Cells were then collected (10,000 rpm, 2 min) and washed with 2 × 600 μl 1xPBS. Cells were fixed in 4% paraformaldehyde in PBS for 20 min. Cells were washed with 2 × 600 μl 1xPBS. Cells were washed 2 × 600 μl 1xPBS, 1 × 200 μl 1xPBS, 1 × 200 μl 1xPBS for 45 min in the dark, and 1 × 200 μl 1xPBS. The cells were resuspended in 100 μl 1xPBS. 15 μl of cells were added to pre-treated coverslips for confocal microscopy. The rest of the cells were stored at 4°C until flow cytometry analysis.

### Bacterial Labeling and Remodeling With CuAAC

Bacterial remodeling and labeling was achieved based on established protocols for *E. coli* (Liang et al., 2017; DeMeester et al., 2019). Briefly, overnight pre-cultured *E. coli ΔMurQ-KU* (EQKU) cells or *P. aeruginosa* were inoculated into fresh LB medium and were incubated until the OD_600nm_ was about 0.600. 1.2 ml of cell suspension were collected by centrifugation at 8,000 rpm (6,000 g) for 5 min. *C*ells were resuspended in 190 μl LB medium. 6mM NAM sugar 1 or 2 (NAM, AzNAM), 1 mM isopropyl-1-thio-β-d-galactoside (*E. coli* only) and 200 μg/ml fosfomycin were added into cell samples. Cells were incubated while shaking at 37°C for 60 min. Cells were then collected (10,000 rpm, 2 min) and washed with 2 × 600 μl 1xPBS. Cells were fixed in 4% paraformaldehyde in PBS for 20 min. Cells were washed with 2 × 600 μl 1xPBS. Cells were resuspended in 190 μl PBS to prepare for the click reaction. To the bioorthogonally tagged bacterial cells was sequentially added 1 mM CuSO_4_ solution, 140 μM BTTAA, 1.2 mM freshly prepared (+)−sodium (L) ascorbate (Sigma-Aldrich) and 20 μM of Alk488. Cells were incubated at room temperature for 30 min. Cells were washed 2 × 600 μl 1xPBS, 1 × 200 μl 1xPBS, 1 × 200 μl 1xPBS for 45 min in the dark, and 1 × 200 μl 1xPBS. The cells were resuspended in 100 μl 1xPBS and prepared for imaging.

### Flow Cytometry for Bacterial Labeling

Flow cytometry was performed on ACEA Novocyte Flow Cytometer. Samples were briefly vortexed before each run. 100,000 cell counts were collected for each sample and were analyzed in triplicate, and fluorescence intensities (height) were generated and overlaid.

### Growth Curve Assay With Plate Reader

Overnight pre-cultured *E. coli ΔMurQ-KU* or *P. aeruginosa* cells were inoculated into fresh LB medium (supplemented with kanamycin and chloramphenicol for *E. coli* cells only) to an OD_600nm_ of approximately 0.200. Cells were incubated at 37°C shaking for ∼1 h 1 ml of cell suspension were added to new sterile Eppendorf tubes and spun at 8,000 rpm for 5 min to pellet the cells. The cells were resuspended in 190 μl of LB and supplemented with 200 μg/ml fosfomycin, 1 mM IPTG for *E. coli* cells only, and 6 mM NAM or AzNAM probe. The cells were then incubated for an additional hour to remodel. Then, the cells were pelleted to remove excess probe at 10,000 rpm for 2 min and resuspended in 100 μl of fresh LB. 100 μl of cell solution was added into each well of a white, clear bottom 96 well plate in triplicate for each probe concentration. 30 μl of DBCO-488 was added to appropriate wells. The plate was incubated at 37°C in the plate reader where it was shaken for 2 min, scanned at 600 nm, and then repeated every 20 min for 6 h (Brown et al., 2021a). Cell growth curves were formulated with Origin 2019.

### Synthesis and Characterization of Norbornene-Functionalized PEG

Multi-arm (Herskovits et al., 2007) PEG (M_n_ ∼ 40,000 g/mol) was functionalized with norbornene end groups according to established protocols (Rehmann et al., 2016; Ovadia et al., 2018). Briefly, in a 250 ml round bottom flask, PEG-8-NH_2_·HCl (5 g, Jenkem) was dissolved in anhydrous *N,N*-dimethylformamide (DMF, ThermoFisher) and stirred at room temperature. In a second 250 ml round bottom flask, 5-norbornene-2-carboxylic acid (Nb-COOH) (17.6 M equivalent; 2.2 excess relative to amine groups on the PEG), 4-methylmorpholine (4-MMP) (36 M equivalent), and HATU (16 M equivalent; 2 excess) were dissolved in 10 ml of DMF stirring at room temperature. Once the individual flask components were dissolved, they were combined into one flask and stirred at room temperature overnight. The solution was then precipitated twice in cold diethyl ether (500 ml, 14x excess diethyl ether relative to DMF), and the resulting suspension was filtered with a Buchner funnel with filter paper to recover the precipitated polymer product. The solid PEG product was dried in the vacuum oven overnight. The PEG was purified by dialysis (MWCO 1000 g/mol, Spectrum Laboratories)) in mqH_2_0 for 48 h according to manufacturer’s instructions followed by freezing and lyophilization. Product purity was confirmed by ^1^H NMR in DMSO-d_6_. Norbornene functionality was determined to be approximately 75% on average (Supplementary Figure S13), and PEG-8-Nb was stored at −20°C following lyophilization.

### Synthesis and Characterization of Peptides

All peptides were synthesized using solid phase peptide synthesis based on established protocols (Ovadia et al., 2018). The difunctional linker peptide (GCRDVPMSMRGGDRCG) and the monofunctional pendant peptide (CGKGYIGSR) were synthesized using standard FMOC-chemistry on an automated peptide synthesizer (PS3 Peptide Synthesizer; Protein Technologies, Inc, Tucson, AZ and Liberty Blue; CEM, Matthews, NC). The peptides were built on Rink Amide MBHA resin. All amino acids were double coupled. The peptides were cleaved from the resin for 2–3 h in 95% trifluoroacetic acid, 2.5% water, and 2.5% triisopropylsilane supplemented with 50 mg/ml dithiothreitol. Following cleavage, all peptides were precipitated in cold diethyl ether at 4°C and let air dry overnight. The peptides were purified by reverse-phase high performance liquid chromatography (HPLC; XBridge BEH C18 OBD 5 μm column; Waters, Milford, MA) with a linear 95%/5 to 5%/95% linear water-acetonitrile (ACN) gradient over 15–30 min. Purified peptides were subsequently lyophilized. Their molecular weights were verified by mass spectrometry (Supplementary Figure S11, Supplementary Figure S12), and the thiol concentration of each peptide upon reconstitution for stock solution preparation was determined using Ellman’s assay. Purified peptides were dissolved in phosphate buffered saline (PBS) and stored at −80°C.

### Synthesis and Rheological Characterization of Hydrogels

Monomer stocks were prepared by dissolving each component in sterile phosphate buffered saline (PBS): PEG-8-Nb (40 mM Nb functionality); lithium phenyl-2,4,6-trimethylbenxoylphospohinate (LAP) (30 mM) sterile filtered with 0.2 µM filter; and each peptide (∼200 mM). PEG-8-Nb and LAP stocks were stored at −20°C, and peptide stocks were stored at −80°C.

A bulk hydrogel precursor solution was prepared using 7 mM PEG-8-Nb, 5 mM peptide crosslinker, 2 mM pendant peptide, and 2 mM LAP in PBS. Rheology measurements were conducted as previously reported (Italiani and Boraschi, 2014) on AR-G2 rheometer with UV-visible light attachment (TA instruments) in tandem with an Omnicure Series 2000 light source (Excelitas) with a 365 nm bandpass filter and light guide (Exfo). Briefly, 10 µl of hydrogel precursor solution was pipetted onto the quartz plate of the UV-vis light attachment on the rheometer with a 8 mm flat plate geometry installed, and the gap was set to 150 µm. Hydrogel crosslinking and gelation were monitored by measuring storage (G′) and loss (G”) moduli at 0.5% applied strain and 2 rad/s frequency upon irradiation (10 mW/cm^2^ at 365 nm). The gelation time was determined to be less than 2 min based on the change in G’ being within 5% between two consecutive points (Geddes et al., 2009). Frequency sweeps at 1% strain were performed after the irradiation was complete to measure the final moduli of hydrogels formed *in situ*. All of the rheometric measurements were performed within the linear viscoelastic regime. Final equilibrium swollen moduli at physiological temperature were calculated as previously reported (Johnston, 1988; Thaiss et al., 2016) using the *in situ* measured modulus and the following equations:

$$G_{final}=G_{0}\left(\frac{T_{final}}{T_{0}}\right){\left(\frac{Q_{final}}{Q_{0}}\right)}^{-\frac{1}{3}}$$ (1)

$$Q_{final}=\left(1-2\chi \right)N^{0.57}\phi^{-0.38}$$ (2)

where G_0_ is the *in situ* measured shear modulus; T_final_ is 310 K; T_0_ is 298 K; Q_0_ is the initial volumetric swelling ratio of the hydrogels; χ is 0.426, the PEG–water interaction parameter; N is 304, the number of PEG repeats between crosslinks; and ϕ is the initial volume fraction of polymer. Lastly, the Young’s modulus (E) was estimated by rubber elasticity theory:

$$E=2G_{final}\left(1+\nu \right)$$ (3)

where ν is Poisson’s ratio and is taken to be 0.5 for these incompressible, elastic PEG hydrogels.

### Mammalian Cell Culture

THP-1, HEK293T, and CCL151 cells were purchased from the American Type Culture Collection (ATCC) and cultured under sterile conditions at 37°C with 5% CO_2_. THP1 cells were grown in RPMI media; HEK293T cells were grown in DMEM media; and CCL151 cells were grown in F12K media. All media was supplemented with 10% FBS (Atlantic Biologicals), 2 mM l-glutamine, and 2 mM penicillin-streptomycin. THP-1 cells were stimulated with 200 nM PMA for 72 h to differentiate into macrophages (Zhang et al., 2008; Daigneault et al., 2010; Lund et al., 2016).

### Cell Encapsulation

THP-1 cells were encapsulated as a single cell suspension at a density of 5 × 10^6^ cells per ml in 20 μl of hydrogel precursor solution (250,000 cells/hydrogel). Precursor solution was prepared using 7 mM PEG-8-Nb, 5 mM linker peptide, 2 mM pendant peptide, 2 mM lithium phenyl-2,4,6-trimethylbenzoylphosphinate (LAP), and THP-1 cell suspension in PBS, where all concentrations noted are for the functional handle. Hydrogels were formed in 10 mm × 0.5 mm sterile gasket molds unless otherwise noted (43 µl precursor solution/mold) upon irradiation with a cytocompatible dose of long wavelength UV light (10 mW/cm^2^, 365 nm, 2 min; Omnicure 2000 with light guide and collimating lens). Two replicates were formed at a time and then placed in a 24 well plate with 500 µl of RPMI media. 200 nM PMA was added to the media in each well for differentiation into macrophages. Cell-hydrogel constructs were incubated under sterile conditions at 37°C with 5% CO_2_.

### Live/Dead Viability Assay on Encapsulated Cells

THP-1 monocyte and macrophage cell viability following encapsulation in the hydrogels was assessed on days 1, 3, and 7 using a LIVE/DEAD^®^ Viability/Cytotoxicity Kit (ThermoFisher Scientific). THP-1 monocytes were encapsulated as a single cell suspension at a density of 5 × 10^6^ cells per ml in 20 μl of hydrogel precursor solution (250,000 cells/hydrogel). Precursor solution was prepared using 7 mM PEG-8-Nb, 5 mM linker peptide, 2 mM pendant peptide, 2 mM LAP, and THP-1 cell suspension in PBS. Hydrogels were formed in 10 mm × 0.5 mm sterile gasket molds (43 µl precursor solution/mold) upon irradiation with a cytocompatible dose of long wavelength UV light (10 mW/cm^2^, 365 nm, 2 min; Omnicure 2000 with light guide and collimating lens). Two replicates were formed at a time and then placed in a 24 well plate with 500 µl of RPMI media. 200 nM TPA was added to the media in each well for differentiation into macrophages. Cell-hydrogel constructs were incubated under sterile conditions at 37°C with 5% CO_2_. Hydrogels (*n* = 3) were removed from incubator on days 1, 3, and 7, and washed 2 × 5 min with 500 μl of PBS followed by a 20-min incubation (37°C at 5% CO_2_) with 500 μL of PBS containing calcein AM (2 μM) and ethidium homodimer-1 (4 μM). After staining, hydrogels were again washed (2 × 5 min with 500 μl of PBS) before imaging. Hydrogels were transferred to a chamber slide (Nunc Lab-Tek™ II Chamber Slide, Glass, 1 well) and imaged with confocal microscopy (Zeiss LSM ×800, ×10 objective at a zoom of ×0.6 and frame size of 1,024 × 1,024 for each image, 200 μm z-stack, three images per hydrogel sample). Orthogonal projections were made of each z-stack, and live (green) and dead (red) cells were counted using ImageJ.

### Flow Cytometry for THP-1 Cell Differentiation

To confirm differentiation of THP-1 cells into macrophages within 3D culture, hydrogels with encapsulated THP-1 cells treated with PMA (for differentiation into macrophages) or untreated THP-1 monocytes were washed twice in 2 ml in 1xPBS for 5 min each. Hydrogels were put into 1.5 ml Eppendorf tubes (4 hydrogels per tube), and a 1 ml solution of collagenase (300 U/ml) (Sigma Aldrich) was added to degrade the hydrogel. Tubes were placed in a CO_2_ incubator at 37°C. The solution was triturated every 10 min for up to 30 min until the solution could be pipetted freely. The now-digested hydrogel solution was centrifuged (150 g, 5 min) to pellet the cells. Monocytes (THP-1 cells) or macrophages (differentiated THP-1 cells) from 2D culture (1 × 10^6^ cells) were removed from plates and similarly centrifuged in 1.5 ml Eppendorf tubes. Pelleted cell samples were washed with 2% bovine serum albumin (BSA) in PBS and resuspended in 100 μl of 2% BSA in PBS, and 5 μl of CD11 b antibody was added. Samples were placed on ice in the dark for 30 min and then centrifuged followed by washing with 2% BSA in PBS. Cells were then fixed in 4% paraformaldehyde in 1xPBS for 15 min at room temperature, centrifuged, and washed 2 × 100 μl in Intracellular Staining Permeabilization Wash Buffer (BioLegend). Cells were resuspended in 100 μl of Intracellular Staining Permeabilization Wash Buffer, and 5 μl of CD68 was added to each sample followed by incubation on ice for 30 min. Cells were washed 1 × 100 μl of Intracellular Staining Permeabilization Wash Buffer. Flow cytometry was performed on ACEA Novocyte Flow Cytometer. Samples were briefly vortexed before each run. 100,000 cell counts were collected for each sample and were analyzed in triplicate, and fluorescence intensities (height) were generated and overlaid.

### DBCO Fluorophore Staining for Fixed Samples

THP-1 cells as monocytes, THP-1 cells differentiated into macrophages using PMA, and HEK293T cells were seeded onto pre-treated coverslips. Cells were fixed with 4% PFA for 10 min at room temperature, and then washed 3 × 5 min with 1x PBS. Cells were then incubated with 30 µM DBCO-488 or an equivalent volume of water as a control for 40 min at 37°C. Cells were washed 3 × 5 min with 1xPBS at room temperature on a rocker. Coverslips were mounted with mounting media with DAPI and imaged using confocal microscopy.

### DBCO Fluorophore Staining for Live Samples

THP-1 cells as monocytes, THP-1 cells differentiated into macrophages using PMA, HEK cells, and CCL151 fibroblast cells were seeded onto pre-treated coverslips. Cells were then incubated with 30 µM DBCO-488 or an equivalent volume of water as a control for 40 min at 37°C. Cells were washed. Cells were fixed with 4% PFA for 10 min at room temperature, and then washed 3 × 5 min with 1x PBS. Coverslips were mounted with mounting media with DAPI and imaged using confocal microscopy.

### Invasion Assay in 2D Culture for Imaging With CuAAC Bacteria

UV sterilized cover glasses (Fisher Scientific, catalogue number 12-545-80) were coated with 500 μl of 0.1 mg/ml poly-L-ornithine (Sigma-Aldrich) in 24-well plate overnight. The poly-L-ornithine was removed, and the cover glasses were washed with PBS twice. THP-1 cells were seeded on the cover glasses in 24-well plates with RPMI media (1 × 10^5^ cells/well). THP-1 cells were differentiated into macrophages through stimulation with PMA for 3 days. Cells were then washed with RPMI media without antibiotics twice. For invasion, *E. coli ΔMurQ-KU* was grown and remodeled following the protocols above for CuAAC. 20 μl of bacterial suspension (OD_600nm_ = 2.0) was added to each well, and the samples were incubated at 37°C and 5% CO_2_ for 30 min. After incubation, the media was removed, and fresh media with gentamycin (1:1,000) was added to kill extracellular bacteria, incubating for 30 min at 37°C and 5% CO_2_. The media then was removed, and the cells were rinsed twice with 1xPBS at room temperature. Cells were fixed 4% paraformaldehyde in 1xPBS for 10 min at room temperature and rinsed twice with 1xPBS. Then, cells were permeabilized with 1% Triton-X in PBS for 10 min at room temperature and washed 3 × 5 min with 1xPBS with 0.2% Tween-20 and 1.5% BSA at room temperature on a rocker. PBS (500 μl) with 0.2% Tween-20 and 0.1% Triton-X was added to each well to prepare for the click reaction. To each well was sequentially added 1 mM CuSO_4_ solution, 140 μM BTTAA, 1.2 mM freshly prepared (+)−sodium (L) ascorbate (Sigma-Aldrich), and 20 μM of Alk488. The click reaction was performed at room temperature for 30 min while shaking. The cells were washed 3 × 5 min with 1xPBS with 0.2% Tween-20 and 1.5% BSA at room temperature on a rocker. The cells were then stained with 1:200 Phalloidin-TRITC (F-actin stain) in 1.5% BSA in 1xPBS for 1 h at room temperature. The cells were washed 3 × 5 min with 1xPBS with 0.2% Tween and 1.5% BSA at room temperature on a rocker and then were mounted on glass slides with 4,6-diamidino-2-phenylindole (Invitrogen) for super resolution imaging.

### Invasion Assay in 2D Culture for Imaging With SPAAC Bacteria

Sterile cover glasses (Fisher Scientific, catalogue number 12-545-80) were coated with 500 μl of 0.1 mg/ml poly-L-ornithine (Sigma-Aldrich) in 24-well plate overnight. The poly-L-ornithine was removed, and the cover glasses were washed with PBS twice. THP-1 cells were seeded on the cover glasses in 24-well plates with RPMI media (1 × 10^5^ cells/well). THP-1 cells were differentiated into macrophages through stimulation with PMA for 3 days. Cells were then washed with RPMI media without antibiotics twice. For invasion, *E. coli ΔMurQ-KU* was grown and remodeled following the protocols above for SPAAC. Following remodeling and labeling with DBCO-488, 20 μl of bacteria (OD_600nm_ = 2.0) was added to each well, and the samples were incubated at 37°C and 5% CO_2_ for 30 min. After incubation, the media was removed, and fresh media with gentamycin (1:1,000) was added to kill extracellular bacteria, incubated for 30 min at 37°C and 5% CO_2_. The media then was removed, and the cells were rinsed twice with 1xPBS at room temperature. Cells were fixed 4% paraformaldehyde in 1xPBS for 10 min at room temperature and rinsed twice with 1xPBS. Then, cells were permeabilized with 1% Triton-X in PBS for 10 min at room temperature and washed 3 × 5 min with 1xPBS with 0.2% Tween-20 and 1.5% BSA at room temperature on a rocker. The cells were then stained with 1:200 Phalloidin-TRITC (F-actin stain) in 1.5% BSA in 1xPBS for 1 h at room temperature. The cells were washed 3 × 5 min with 1xPBS with 0.2% Tween and 1.5% BSA at room temperature on a rocker and then were mounted on glass slides with 4,6-diamidino-2-phenylindole (Invitrogen) for confocal microscopy imaging.

### Invasion Assay in 3D Culture for Imaging With CuAAC Bacteria

THP-1 cells were encapsulated with an adapted version of the protocol described above. Here, cells suspended in hydrogel precursor solution were prepared as before, and then 20 μl of hydrogel precursor solution was pipetted onto a 1 ml syringe mold (instead of a gasket mold) resulting in 100,000 cells per hydrogel. Hydrogels were formed by photopolymerization and placed into 24-well plates for 3D cell culture and differentiation with PMA for 3 days as before. Samples were then washed with RPMI media without antibiotics twice. For invasion, 20 μl of *E.* *coli ΔMurQ-KU* (OD_600nm_ = 2.0), grown and remodeled as noted above for the CuAAC protocol, was added to each well for 60 min, and the samples were incubated at 37°C and 5% CO_2_. After incubation, the media was removed, and fresh media with gentamycin (1:1,000) was added to kill extracellular bacteria for 60 min at 37°C and 5% CO_2_. After 60 min, the media was removed, and the cells were rinsed twice with 1 x PBS at room temperature. Cells were fixed 4% paraformaldehyde in 1xPBS for 15 min at room temperature. Fixed cells were rinsed 3 × 5 min with 1xPBS, and were permeabilized with 1% Triton-X in PBS for 30 min at room temperature. Cells were washed 3 × 5 min with 1xPBS with 0.2% Tween-20 and 1.5% BSA at room temperature on a rocker. PBS (500 μl) with 0.2% Tween-20 and 0.1% Triton-X was added to each well to prepare for the click reaction. To each well was sequentially added 1 mM CuSO_4_ solution, 128 μM Tris [(1-benzyl-1H-1,2,3-triazol-4-yl)methyl]amine, 1.2 mM freshly prepared (+)−sodium (L) ascorbate (Sigma-Aldrich), and 20 μM of Alk488. The click reaction was performed at room temperature for 1 h while shaking. The cells were washed 2 × 30 min with 1xPBS with 0.2% Tween-20 and 1.5% BSA at room temperature on a rocker. The cells were washed overnight in 1xPBS with 0.2% Tween-20 and 1.5% BSA at 4°C. The next day, the cells were washed 2 × 30 min with 1xPBS with 0.2% tween-20 and 1.5% BSA at room temperature on a rocker. Cells were stained with 4,6-diamidino-2-phenylindole (DAPI) for 30 min and were washed 3 × 10 min with 0.2% Tween-20 and 1.5% BSA at room temperature on a rocker. Hydrogels were moved to glass chamber well slides for confocal imaging.

### Invasion Assay in 3D Culture for Imaging With SPAAC Bacteria

THP-1 cells were encapsulated as described above. Hydrogels were formed by photopolymerization and placed into 24-well plates for 3D cell culture and differentiation with PMA for 3 days as before. Samples were then washed with RPMI media without antibiotics twice. For invasion, 20 μl of *E. coli ΔMurQ-KU* (OD_600nm_ = 2.0), grown and remodeled as noted above with the SPAAC protocol, was added to each well for 60 min, and the samples were incubated at 37°C and 5% CO_2_. After incubation, the media was removed, and fresh media with gentamycin (1:1,000) was added to kill extracellular bacteria for 60 min at 37°C and 5% CO_2_. After 60 min, the media was removed, and the cells were rinsed twice with 1xPBS at room temperature. Cells were fixed 4% paraformaldehyde in 1xPBS for 15 min at room temperature. Fixed cells were rinsed 3 × 5 min with 1xPBS, and were permeabilized with 1% Triton-X in PBS for 30 min at room temperature. Cells were washed 3 × 5 min with 1xPBS with 0.2% tween-20 and 1.5% BSA at room temperature on a rocker Cells were stained with DAPI for 30 min and were washed 3 × 10 min with 0.2% Tween-20 and 1.5% BSA at room temperature on a rocker. Hydrogels were moved to glass chamber well slides for confocal imaging.

### Live Cell Imaging

THP-1 cells were seeded into 8-well chamber slides and treated with 20 nM PMA to differentiate for 3 days. Media was replaced to RPMI without antibiotics. Cells were then incubated with Cell Mask Orange for 15 min. The media was changed and SPAAC labeled bacteria (see Bacterial Labeling and Remodeling) with strain promoted azide-alkyne cycloaddition (SPAAC) was added to the appropriate wells. The cells were imaged using the Andor Dragonfly 505 spinning disk confocal microscope with a Plan-Apochromat 63x/1.47 oil objective to visualize the engulfment of bacteria cells in real time. Excitation of labeled bacteria and Cell Mask Orange was achieved with a 637 nm laser at 2% power and a 561 nm laser at 0.2% power. 3D volumes were acquired every 1 min for 2 min.

### Confocal Microscopy for 2D Samples

*P. aeruginosa* images and 2D invasion images were taken on a Zeiss LSM 800 microscope with Plan-Apochromat 63x/1.4 Oil differential interference contrast (DIC) M27 objective. Excitation of DBCO-488 was achieved with 488 nm, 2% laser excitations. Excitation of phalloidin-TRITC was achieved with 562 nm, 0.2% laser excitations. Excitation of DAPI was achieved with 405 nm, 1% laser excitation. Scan mode was frame and bidirectional. Program Carl Zeiss ZEN 2012 was used to process the raw data to construct the images. Processing and filtering settings were kept constant and image intensity was preserved with the raw image scale option in Zen 2012. Two-dimensional (2D) images were generated. Scale bars were made with the line measurement tool function.

### Structured Illumination Microscopy (SIM) for 2D Samples

Bacterial labeling of EQKU cells was imaged on a Zeiss Elyra PS.1 microscope with Plan-Apochromat 63x/1.4 oil differential interference contrast (DIC) M27 objective. Excitation of Alk488 was achieved with 488 nm laser excitation, and the camera exposure time was set to 100.0 ms. The raw data contained five rotations with 0.110 μm z-stack interval. Images were processed in Carl Zeiss ZEN 2012 to construct SIM images. Processing and filtering settings were kept constant and image intensity was preserved using the raw image scale option. Two-dimensional (2D) SIM images and 2D maximum intensity projection images were generated.

### Confocal Microscopy Imaging for 3D Samples

3D culture samples were imaged with confocal microscopy. Images were taken on Zeiss LSM800 with Plan-Apochromat 63X/1.40 Oil DIC M27 objective and frame size of 1,024 × 1,024 pixels. Z-stacks were 200 μm with 0.2 μm slices. Excitation of 4,6-diamidino-2-phenylindole and Alk488 was achieved with 405 and 488 nm lasers, respectively. Pixel, line, and frame time were 1.03 μs, 4.95 ms, and 5.06 s, respectively. Scan direction was bidirectional, and an average of four scans per image was utilized. ZEN 2012 (Zeiss) was used to process the images and prepare z-stack projections.

### ELISA Preparation

Three days before stimulation with bacteria, macrophage samples were seeded and encapsulated for 2D and 3D culture, respectively. For 2D culture, cells were seeded at 1 × 10^6^ cells per well in a 6 well plate. For 3D culture, hydrogels were formed in gasket molds as described above, and four hydrogels (250,000 cells per hydrogel) were placed per well in a 6 well plate (total of 1 × 10^6^ cells per well). All cells were differentiated for 3 days with 200 nM PMA. The cells were washed twice with RPMI media without antibiotics. Cells were provided RPMI media without antibiotics after the washes. The day of the stimulation with bacteria, bacterial overnights were diluted to OD = 2.0, and 20 μl; of these bacteria was added to each well. To the control samples, 20 μl of sterile water was added. Plates were incubated for 4 h in an incubator (37°C, 5% CO_2_). After 4 h, the supernatant was removed, and it was stored at −20°C until shipment (1 ml total volume per sample) to University of Maryland Cytokine Core for analysis by ELISA.

## Results and Discussion

### Bacterial Remodeling and Labeling Utilizing CuAAC and SPAAC

**FIGURE 2:**
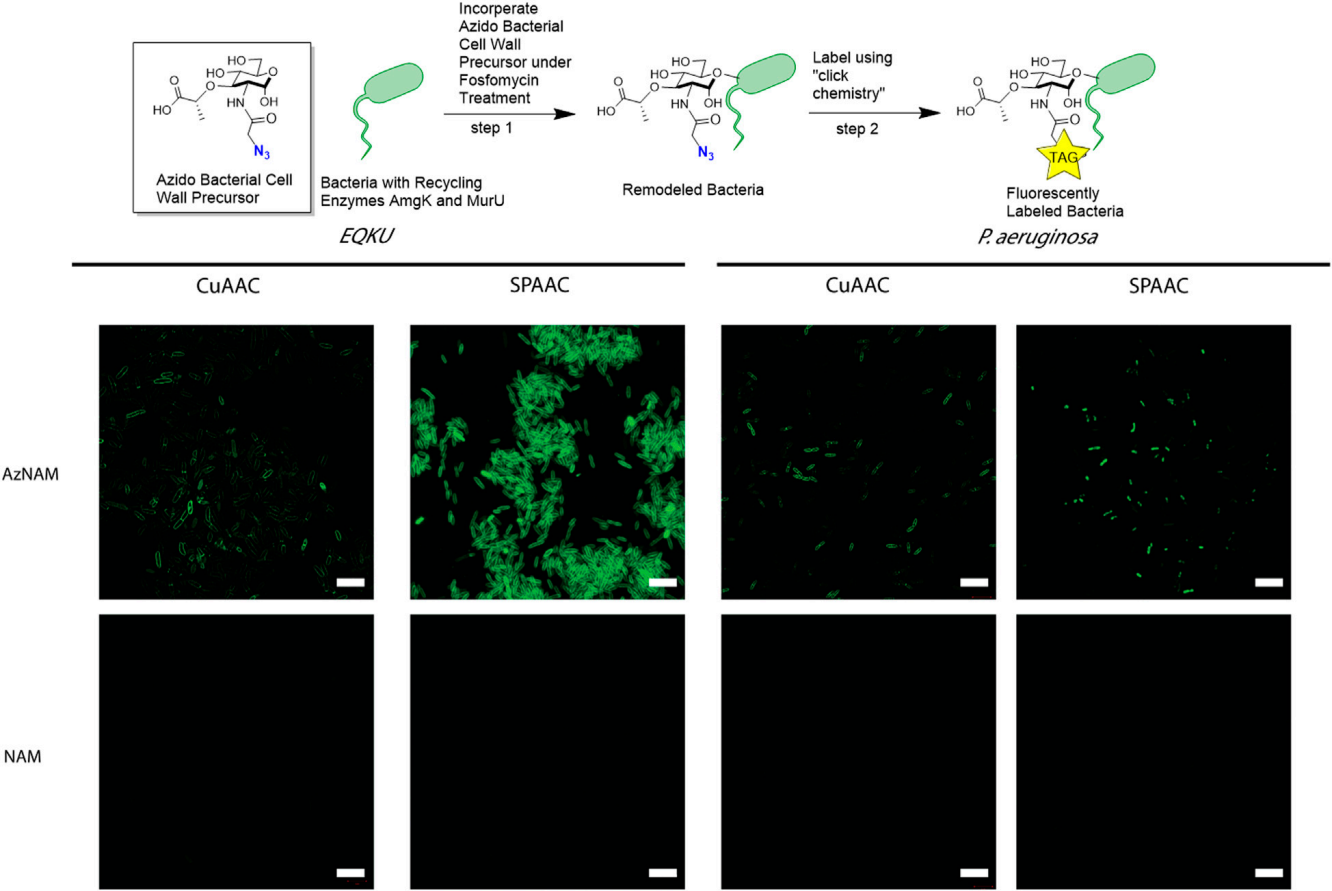
Labeling of Gram-negative bacteria utilizing CuAAC and SPAAC chemistries. *E. coli ΔMurQ KU* (EQKU) and *P. aeruginosa* cells are remodeled by incorporating the azido bacterial cell wall precursor (AzNAM) in the cell wall with recycling enzymes AmgK and MurU. Bacteria are then fluorescently labeled using CuAAC or SPAAC to install a terminal alkyne (CuAAC) or strained alkyne (SPAAC) fluorophores. NAM cells do not have the azide handle and therefore do not react with the fluorophores, leading to no fluorescence. CuAAC images were taken using super resolution microscopy (SIM) and SPAAC images were taken using confocal microscopy. Images are representative of a minimum of three fields viewed per replicate with at least two technical replicates, and experiments were conducted in at least three biological replicates. Scale bars = 10 µM.

A previously engineered strain of *E. coli*, *E. coli ΔMurQ KU* (EQKU) that expresses recycling enzymes AmgK and MurU was used to specifically label the NAM residue of PG (Liang et al., 2017). Through remodeling with the 2-azido *N*-acetyl muramic acid sugar (AzNAM), we observed fluorescent labeling specifically to the cell wall of the EQKU PG following the copper catalyzed azide-alkyne cycloaddition (CuAAC) click reaction to install an alkyne fluorophore (Alk488) which matches previously reported data from our lab (Figure 2).

As the *Pseudomonas* family naturally express AmgK and MurU, we sought to expand our CuAAC labeling methodology beyond EQKU into the pathogenic species *Pseudomonas aeruginosa,* an opportunistic, pathogenic species of bacteria that is known to break through mucosal barriers particularly in hospital infections and is extremely antibiotic resistant (Lister et al., 2009). Previous work has shown success in applying this CuAAC methodology to *P. putida*, *B. subtilis*, and *H. pylori* (Liang et al., 2017; Taylor et al., 2020; Taylor et al., 2021). Once again utilizing the AzNAM probe and a lethal dose of the cell wall targeting antibiotic, fosfomycin, the first steps of PG biosynthesis are inhibited and we observed incorporation of the AzANM probe using AmgK and MurU recycling enzymes into the *P. aeruginosa* cell wall as confirmed by fluorescent microscopy following CuAAC click reaction (Figure 2).

**FIGURE 3:**
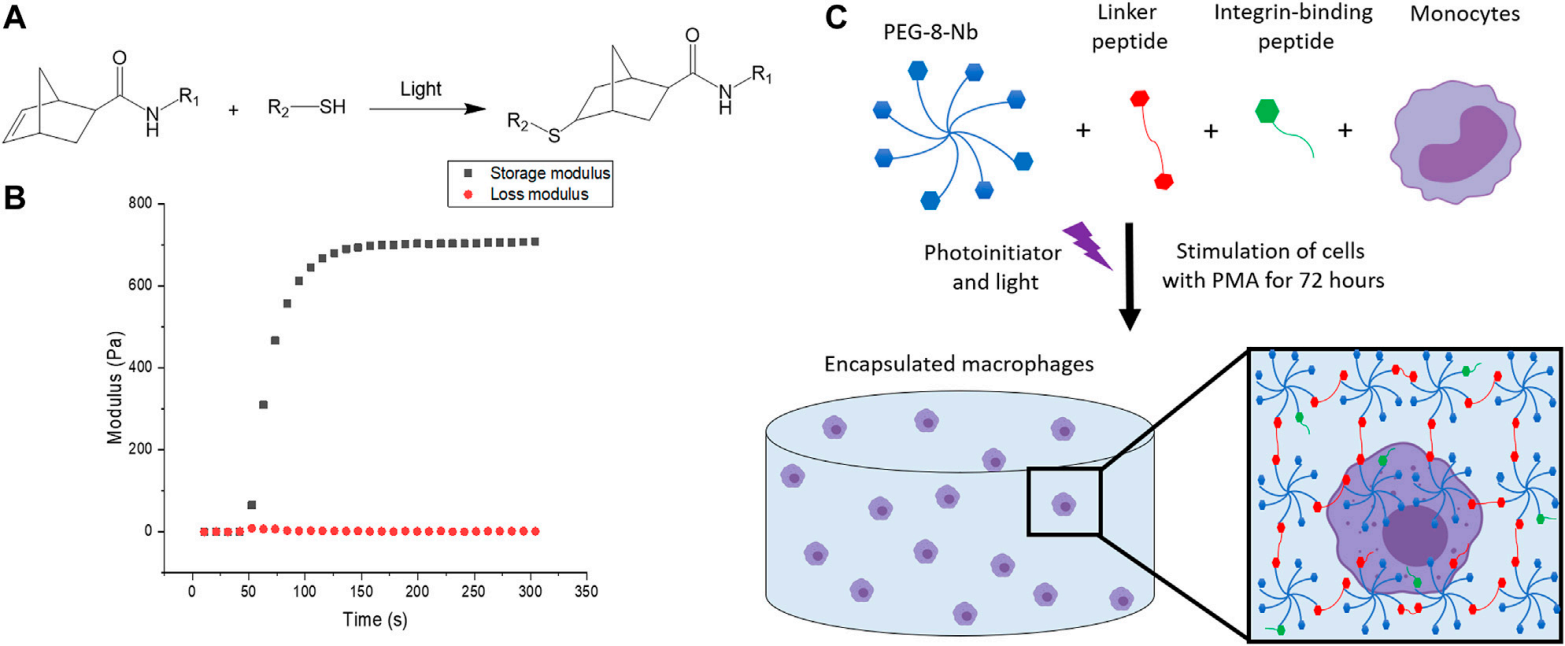
Overview of hydrogel design. **(A)** Hydrogels were formed using thiol-ene click chemistry with norbornenes on the PEG and thiols on the difunctional linker and monofunctional integrin binding peptides. **(B)** Hydrogels are rapidly formed upon the application of light. The increase in storage modulus, monitored with *in situ* rheometry, indicates crosslinking events, and complete hydrogel formation was observed within 2 min. This final modulus, converted to Young’s modulus by rubber elasticity theory for ease of comparison to native tissues, is relevant for mimicking the modulus or “stiffness” of the healthy gut. **(C)** The hydrogel network is composed of 8-arm PEG-norbornene (blue), thiol-containing linker peptide (red), and thiol-containing integrin binding peptide (green). Components were photo-polymerized with a low, cytocompatible dose of light (10 mW/cm^2^ at 365 nm for 2 min) to form a hydrogel, and the monocyte cells in the hydrogel subsequently were differentiated into macrophages through stimulation with PMA for 72 h. Schematic not to scale.

Although the CuAAC click reaction allows for the visualization of the cell wall, reaction requires the cells to be fixed due to the toxic nature of copper to cells, eliminating the ability of tracking the bacteria in real time to get engulfed by macrophages. Therefore, we explored another bioorthogonal reaction called the strain promoted azide-alkyne cycloaddition (SPAAC) that does not require a cytotoxic catalyst, allowing for the possibility of live cell imaging (Baskin et al., 2007). Some applications of SPAAC include labeling proteins, lipids, glycans on mammalian cells (Neef and Schultz, 2009; Nikić et al., 2015; Debets et al., 2020), in living animals such as mice (Chang et al., 2010), ribonucleic acid (RNA) (Wang et al., 2020), and more recently, on bacteria using penicillin binding proteins (Brown et al., 2021b). Since we previously remodeled both *E. coli* and *P. aeruginosa* with AzNAM, we utilized a dibenzocyclooctyne (DBCO) 488 dye as our strained alkyne. Through a detailed study of DBCO concentrations and click reaction times, we determined that 40 min at 37°C with 30 μM DBCO-488 was sufficient to label the cell wall in *E. coli* for microscopy (Figure 2, Supplementary Figure S1), demonstrating that the DBCO dye can cross the outer membrane of the bacterial cell. Flow cytometry revealed that a concentration higher than 30 μM DBCO leads to a secondary population of unlabeled cells in *E. coli*. Interestingly, the SPAAC reaction on AzNAM remodeled *P. aeruginosa* appeared to be located predominantly in the poles and leads to a shorter cell length than CuAAC labeled *P. aeruginosa* (Figure 2). A plate reader growth curve assay was utilized to observe how growth rates of species were different following the click reaction on the living cells and found that both cell type’s growth rates were unaffected by the chemistry occurring on the cell wall (Supplementary Figure S2, Figure 3). We hypothesize that differences in cell wall architecture between the two species lead to the modification being more permittable in *E. coli* and is causing the probe to get stuck in the poles of the *P. aeruginosa* cells. With the CuAAC and SPAAC reactions working well in *E. coli*, we proceeded with this specific species to visualize engulfment by macrophages in both 2D and 3D invasion models.

### Development of 3D Synthetic Matrix for Macrophage Culture

To fabricate a 3D synthetic matrix with tunable properties in the range of those of the healthy gut, we utilized an 8-arm PEG functionalized with norbornene end groups (PEG-8-Nb) linked with a matrix metalloproteinase (MMP) - degradable sequence (GCRDVPMS↓MRGGDRCG) (Ovadia et al., 2018) that is responsive to MMP-2 amongst other enzymes secreted by monocytes and macrophages (Ahmad et al., 2019; Chellat et al., 2005). Additionally, these hydrogels were modified with the integrin binding peptide CGKGYIGSR, derived from the laminin *β*_1_ chain, to promote cell adhesion inspired by the laminin-rich ECM of the basement membrane of the gut (Smithmyer et al., 2014; Yamada et al., 2011; Rezakhani et al., 2021). The hydrogels were formed rapidly through light-triggered thiol-ene click chemistry *via* a step growth mechanism, as the norbornene groups on the PEG are coupled to the thiols presented by the cysteines in the difunctional MMP-degradable linker peptide and monofunctional integrin binding peptide (Figures 3A,B) (Fairbanks et al., 2009). The reaction was initiated using the photoinitiator lithium phenyl-2,4,6-trimethylbenzoylphosphinate (LAP) and low, cytocompatible doses of light (10 mW/cm^2^ at 365 nm for 2 min). These synthetic bioinspired hydrogels were designed with a Young’s modulus (E ∼ 2.6 ± 0.8 kPa) to mimic the “stiffness” of the healthy gut (Johnson et al., 2013), as confirmed by shear rheometry (Figure 3C).

**FIGURE 4:**
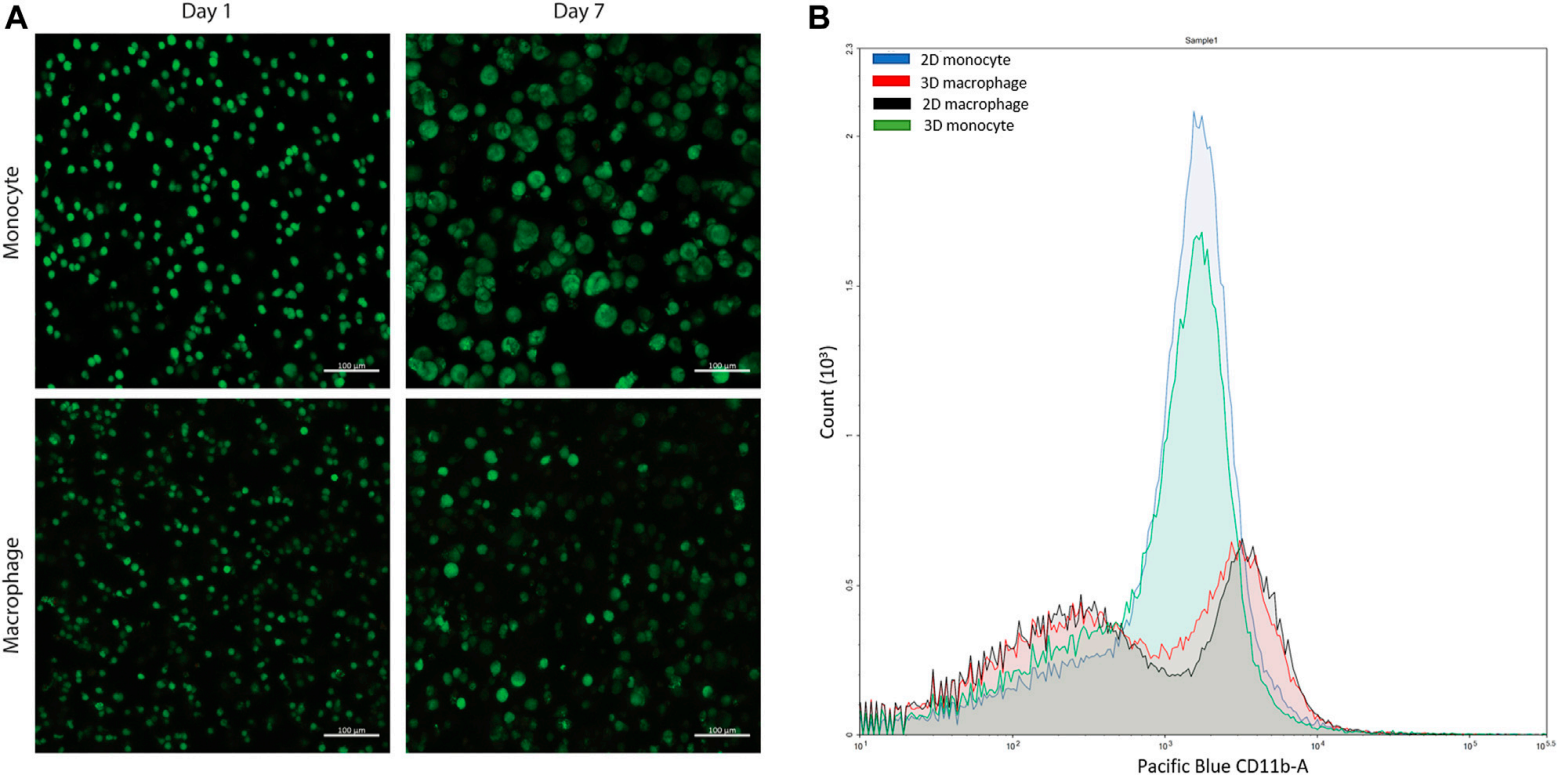
Confirmation of cell viability and cell differentiation in 3D culture. **(A)** Live/dead cytotoxicity results of THP-1 cells (monocytes) and THP-1 cells differentiated with PMA (macrophages) in PEG-peptide hydrogels. Confocal images are representative of a minimum of three fields per view per hydrogel of three biological replicates. **(B)** Flow cytometry data of macrophages and monocytes in 2D or 3D culture stained for extracellular macrophage marker CD11 b. 2D (blue) and 3D monocyte (green) profiles match each other, and 2D (red) and 3D (black) macrophage profiles match each other, showing differentiation is achieved in the 3D hydrogel. Plot is representative of three biological replicates and two technical replicates.

This type of PEG-peptide hydrogel, formed by a photoinitiated thiol-ene step growth polymerization under similar conditions (10 mW/cm^2^ at 365 nm for < 10 min), has been used previously to culture a wide variety of cell types, including human mesenchymal stem cells, induced pluripotent stem cells, and breast cancer cells (Tibbitt and Anseth, 2009; Caliari and Burdick, 2016; Ovadia et al., 2018; Gerecht et al., 2007; Benoit et al., 2008). However, as the mechanism of hydrogel polymerization involves free-radicals, the viability of sensitive cell types can be impacted during cell encapsulation and hydrogel formation (Caliari and Burdick, 2016; Ovadia et al., 2018; Macdougall et al., 2018). Accordingly, we examined cell viability upon encapsulation and during 3D cell culture within these materials. Here, THP-1 cells were selected as a human monocytic cell line that are commonly differentiated into macrophage cells, as human primary tissue macrophages cannot be readily expanded *ex vivo* (Daigneault et al., 2010)*.* We first differentiated monocytes into macrophages on TCPS and subsequently encapsulated these cells. We observed low viability in the hydrogels (Supplementary Figure S4). Therefore, we examined encapsulating monocytes and then differentiating them into macrophages within the matrix, which also mimics aspects of the natural process of monocyte arrival and differentiation into macrophages within the native gut. Importantly, monocytes were successfully encapsulated within the hydrogels and exhibited high viability throughout 3D culture (Figure 4A, Supplementary Figure S5). Based on observations from prior studies with other human cell types, we speculate that the cytotoxic effects of hydrogel formation on macrophages were related to their sensitivity to free radical exposure (Caliari and Burdick, 2016; Macdougall et al., 2018; Ovadia et al., 2018); opportunities for future investigations include examination of the specific cause(s) of macrophage death in this system and related approaches for rescuing human macrophage cell viability (e.g., inclusion of different ligands for promoting cell health (Ovadia et al., 2018), examination of different photoinitiators or irradiation wavelengths (Bryant et al., 2000; Williams et al., 2005; Zeng et al., 2021), or use of a different hydrogel formation mechanism (Macdougall et al., 2018). For the purpose of our studies here, focused on probing bacterial cell-macrophage interactions, the system established functioned well for maintaining monocyte viability and allowing their differentiation into macrophages during 3D culture, which has broad utility for probing not only macrophage-bacteria interactions but also monocyte-bacteria interactions in future studies.

After confirmation of successful culture of THP-1 monocyte cells, monocytes were differentiated into macrophage cells through stimulation with phorbol 12-myristate 13-acetate (PMA) over 3 days based on literature precedent in two dimensions (Zhang et al., 2008; Daigneault et al., 2010; Lund et al., 2016). To probe differentiation, the hydrogels were digested using collagenase and centrifuged to isolate the cells from the materials to study changes in their extracellular and intracellular macrophage markers. Through subsequent staining and flow cytometry, differentiation was confirmed for the recovered cells using macrophage extracellular and intracellular markers, CD11 b and CD68, respectively (Figure 4B, Supplementary Figure S6). This hydrogel digestion method can be applied to isolate a wide variety of 3D cultured mammalian cells for downstream flow cytometry analysis. Further, these macrophages also exhibited high viability in the hydrogels (Figure 4A, Supplementary Figure S5).

### Invasion of Fluorescent Bacteria Into Macrophages in 2D and 3D Culture

**FIGURE 5:**
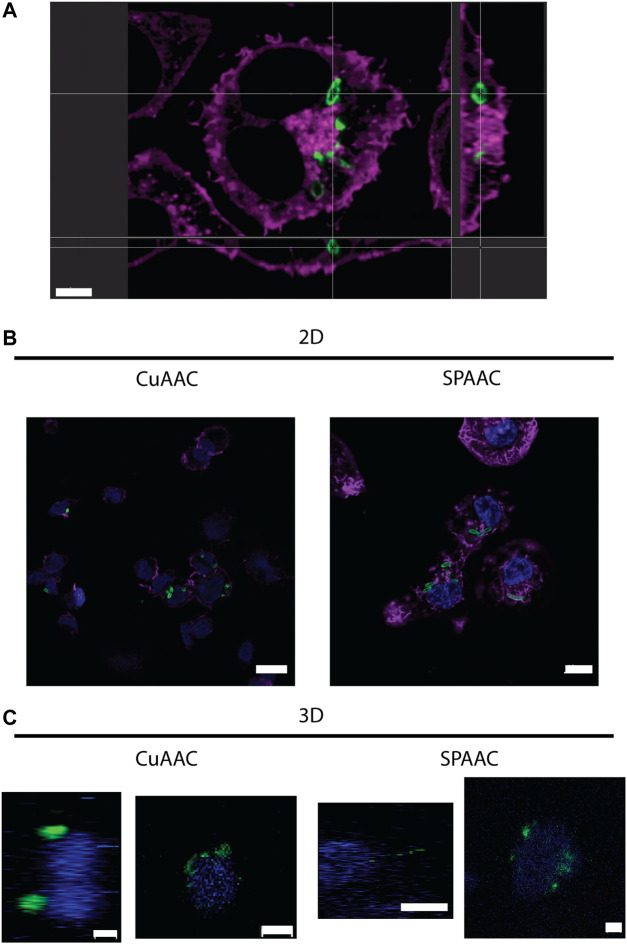
THP-1 macrophage cells invaded by remodeled *E. coli ΔMurQ KU* in 2D and 3D culture. *E. coli ΔMurQ KU* (EQKU) cells are remodeled by incorporating the azido bacterial cell wall precursor in the cell wall with recycling enzymes AmgK and MurU. **(A)** EQKU cells labeled with DBCO-488 using SPAAC were invaded into macrophage cells and observed with Andor Dragonfly microscope. Bacteria (green) can be visualized within macrophages (purple) as shown by ortho view above. Scale bar = 5 μm **(B,C)** The CuAAC condition bacteria were invaded into THP-1 macrophage cells and fixed prior to the CuAAC click labeling (bacteria, green) and subsequent staining with Hoechst (nucleus, blue). The SPAAC condition bacteria were remodeled with the DBCO-488 (bacteria, green) and then invaded into the THP-1 cells. The cells were then fixed and stained with Hoechst (nucleus, blue). The 2D samples **(B)** were also stained with Phalloidin-TRITC (F-actin, purple). Images were taken on a Zeiss LSM800 Confocal Microscope with single plain snaps for 2D images and ortho-projections (XYZ) of Z-stacks of the cells in the synthetic hydrogels. Images are representative of a minimum of three fields viewed per replicate with at least two technical replicates, and experiments were conducted in at least three biological replicates. **(B)** Scale bars = 10 μm **(C)** Scale bars = 5 μm.

With the ability to fluorescently label *E. coli* with CuAAC and SPAAC, macrophages were invaded with bacteria on 2D culture on TCPS and into 3D cultures within synthetic hydrogels. For the CuAAC click reactions, EQKU were remodeled with AzNAM and subsequently invaded into macrophages, followed by fixation and the CuAAC reaction. After invasion for 1 h, we observed engulfment of the bacteria by the macrophages in both 2D and 3D culture (Figure 5), indicating that this system will have utility in identifying naturally released bacterial peptidoglycan fragments. We also noted that the bacteria in 3D culture invasion assay appeared to be more similar in size to the expected biologically-relevant lengths of 1–2 μm, whereas bacteria were larger in 2D invasion (Ei-Hajj and Newman, 2015). Overall, these observations suggest that the dimensionality of the culture system affects rate of bacterial breakdown to smaller fragments and bacterial growth independently of macrophages in these types of 3D materials. We also remodeled *E. coli* and labeled with SPAAC prior to invasion into macrophages. Following fixation and staining, we can observe EQKU within the macrophages in 2D and 3D culture (Figure 5). This pre-labeling method of the bacteria with SPAAC is also advantageous as it allows us to visualize the engulfment in real time. Within 5 minutes in live samples, we can observe engulfment of EQKU by macrophages in 2D culture (Figure 5, Supplementary Videos S1, S2).

Next, we wanted to determine if remodeled EQKU can be invaded into macrophages and a subsequent SPAAC click chemistry can be performed once the bacteria are already inside of the cell; this would mimic the CuAAC labeling method. Following the engulfment of the bacterial cells, the macrophage cells were fixed and then treated the cells with DBCO-488 to label the bacteria in the system. The confocal microscopy images revealed the labeled of EQKU cells but also significant, non-specific binding of the fluorophore to the cells (Supplementary Figure S7) despite rigorous washing steps. To determine the cause of the background dye labeling, macrophage cells were treated as well as monocytes (undifferentiated THP-1 cells) and HEK293T cells with 30 μM DBCO-488 after fixing the cells. We determined that the utilization of a fixative agent causes the DBCO dye to nonspecifically bind to the macrophage cells as well as THP-1 monocyte cells and HEK293T cells; this behavior has been observed in the literature (Supplementary Figure S8) (Loebel et al., 2019). Interestingly, when treating live cells with the DBCO dye prior to fixation, only the macrophage cells continue to have nonspecific binding while THP1 monocyte cells, HEK293T, and CCL151 fibroblast cells do not nonspecifically label (Supplementary Figure S9). We suggest that this is due to the phagocytic and pinocytotic nature of macrophage cells to continuously sense their local environment for possible pathogens. We caution those culturing macrophages to find alternatives to DBCO dyes for fixed SPAAC staining.

### Cytokine Screen From Macrophages Following Bacterial Invasion in 2D and 3D

Following successful invasion of macrophages in both 2D and 3D culture, we aimed to probe potential differences in cytokine expression as a measure of macrophage activation upon invasion, which may be influenced by the dimensionality and complexity of the culture system. Here, we utilized EQKU and *P. aeruginosa,* both unmodified. To assess macrophage response, we examined secretion of cytokines associated with different aspects of macrophage activation. Specifically, an enzyme-linked immunosorbent assay (ELISA) was implemented to observe cytokine output of tumor necrosis factor alpha (TNF-α) and interleukin 6 (IL-6) of cells treated with *E. coli*, *P. aeruginosa*, or no bacteria. TNF-α is a cytokine involved in systemic inflammation and regulates immune cells, induces fever, inhibits viral replication, and induces apoptosis. Dysregulation of TNF-α is implicated in various diseases including IBD. IL-6 is a cytokine involved in stem cell differentiation, antibody synthesis by B cells, and T cell cytotoxicity (Sanceau et al., 1991). IL-6 is produced in response to bacterial and viral infections, and work has shown that dysregulation of IL-6 can be involved in autoimmune disease pathogenesis (Sanceau et al., 1991).

**FIGURE 6:**
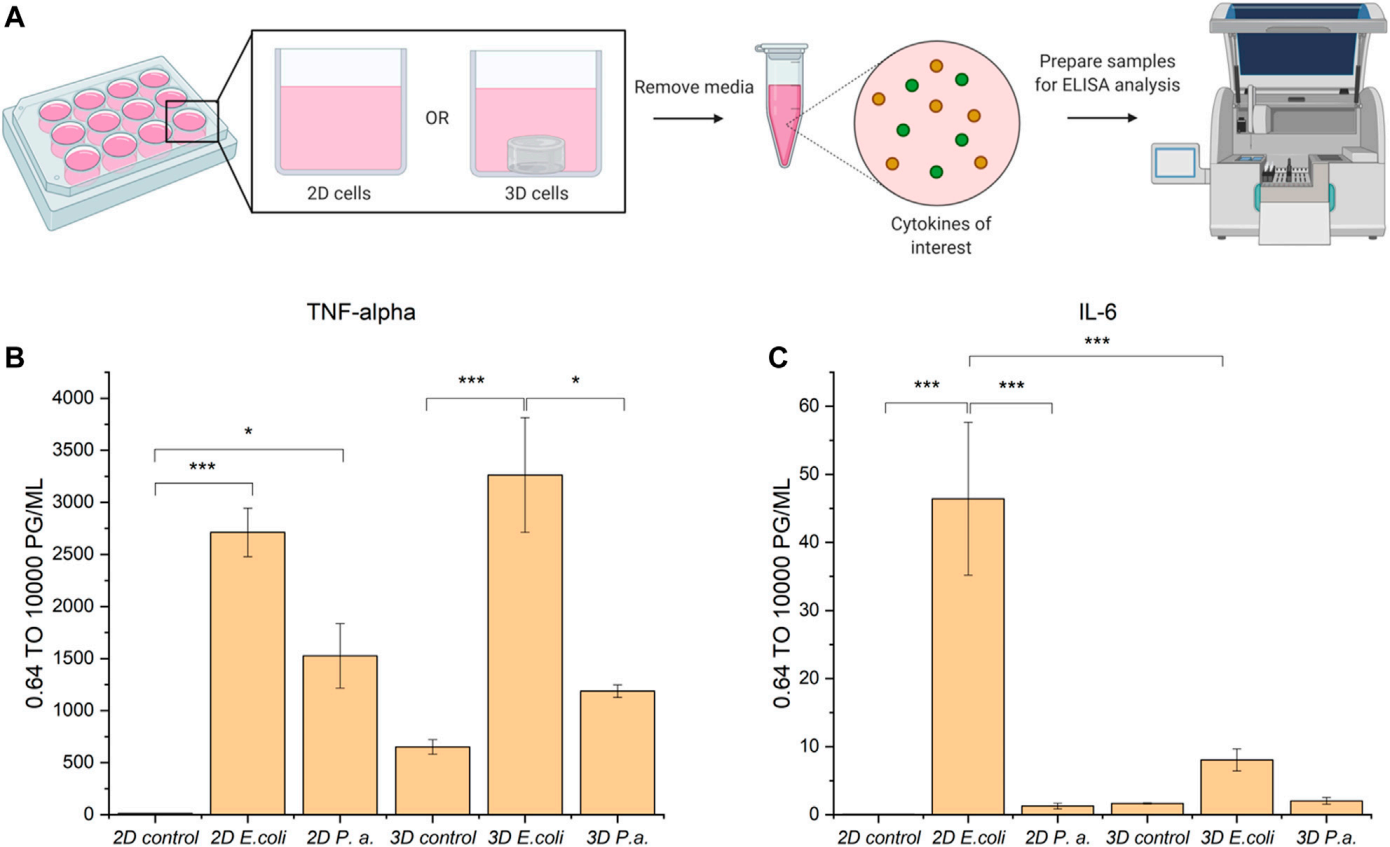
ELISA analysis of TNF-α and IL-6 cytokine production by THP-1 macrophage cells in 2D and 3D culture upon invasion with *E. coli* or *P. aeruginosa*. **(A)** Macrophage cells in 2D or 3D culture were stimulated with bacteria or water for 4 h. Cell culture supernatant was harvested and subjected to ELISA analysis for TNF-alpha and IL-6 cytokines. **(B)** ELISA analysis of TNF-alpha shows cells stimulated with *E. coli* or *P. aeruginosa* exhibited increased secretion of TNF-alpha as compared to the control. **(C)** ELISA analysis of IL-6 shows cells stimulated with *E. coli* in 2D exhibited increased secretion of IL-6 as compared to in 3D culture and the controls. Macrophages in 2D and 3D culture when stimulated with *P. aeruginosa* did not exhibit increased secretion of IL-6 as compared to the control. All samples were analyzed in biological triplicate and technical duplicate and statistically analyzed by ANOVA with Tukey’s test (*p < 0.05, **p < 0.01, ***p < 0.001).

When macrophages were invaded with either *E. coli* or *P. aeruginosa*, an increase in TNF-α expression was observed*,* which accurately represents known responses that THP-1 macrophages have to lipopolysaccharide (LPS) located on Gram-negative bacteria (Figure 6) (Zhang et al., 2008; Schildberger et al., 2013). Further, THP-1 macrophage cells from 2D plates treated with *E. coli* showed statistically significant increased expression of IL-6 as compared to untreated cells and cells treated with *P. aeruginosa*. Notably, THP-1 macrophage cells treated with *E. coli* in 3D culture did not show statistically different expression of IL-6 compared to the control and *P. aeruginosa* samples (Figure 6). These data suggest that the 3D invasion model produced a more biologically relevant response of IL-6 to the various bacterial species, as literature precedent shows THP-1 macrophages treated with LPS are known to have little IL-6 response (Schildberger et al., 2013).

## Conclusion

Overall, we have established a well-defined hydrogel-based system for the 3D culture of monocytes and macrophages in an environment that mimics aspects of the basement membrane of the gut, and a multi-dimensional invasion assay for probing macrophage response to invading bacterial species. The multiscale system allows the integration of probes within the bacterial cell wall at the molecular level, control of bacterial-host interactions at the cellular level, and mimicry of aspects of the ECM dimensionality, biochemical content, and biophysical properties at the multi-cellular to tissue level initially and over time. This molecularly engineered system is reproducible, robust, and based on the underlying design of the synthetic matrix, provides future opportunities for not only probing the impacts of host-bacterial cell interactions, but also cell-matrix interactions through manipulation of extracellular biochemical and mechanical cues. The system is amenable to bioorthogonal chemistry on bacterial peptidoglycan within the hydrogel and opens the door for further applications in chemical biology involving labeling and study of the bacterial cell wall. This model system can be easily tailored for studying cell-cell and cell-matrix interactions in disease progression and for asking new questions beyond traditional 2D culturing methods that prevail in the field. The use of the NAM probes with the azide bioorthogonal handle embedded in the bacterial peptidoglycan allows for multiple click chemistry to take place, including CuAAC and SPAAC, and will provide opportunities to enrich for biologically produced bacterial cell wall fragments from these invasion systems for further analysis. These studies provide new insights into the interactions between bacteria and immune cells, and this new chemical biology model system provides a platform to examine how our immune system recognizes bacteria in a variety of physiologically relevant settings, including pathogen invasion and homeostatic maintenance.

## Data Availability

The raw data supporting the conclusion of this article will be made available by the authors, without undue reservation.
