# Supplementary material for: Multiscale Invasion Assay for Probing Macrophage Response to Gram-Negative Bacteria
Source: Front Chem. 2022 Feb 15;10:842602. doi: 10.3389/fchem.2022.842602 (PMC8886205; doi:10.3389/fchem.2022.842602)
Supplement: Supplementary file 3 [file DataSheet1.docx]

**Multiscale Invasion Assay for Probing Macrophage Response to Gram-Negative Bacteria**

Kimberly A. Wodzanowski^1^, Jeffrey L. Caplan^2,3^, April M. Kloxin^4,5^*, and Catherine L. Grimes^1,2^*

^1^Department of Chemistry and Biochemistry, University of Delaware, Newark, DE 19716

^2^Department of Biological Sciences, University of Delaware, Newark, DE 19716

^3^Bioimaging Center, Delaware Biotechnology Institute, Newark, DE 19716

^4^Department of Chemical and Biomolecular Engineering, University of Delaware, Newark, DE 19716

^5^Department of Materials Science and Engineering, University of Delaware, Newark, DE 19716

*co-corresponding authors

**Table of Contents**

1. **General Materials and Methods…………………………………………………….… 3**
2. **Concentration and Reaction Time Screen of SPAAC Reaction on *E. coli* Peptidoglycan…………………………………………………………………………....4**
3. **Growth curve analysis of SPAAC Reaction of EQKU……….…………………….…5**
4. **Growth Curve Analysis of SPAAC Reaction of *P. aeruginosa*…..……...………….…6**
5. **Live/Dead of Macrophages Differentiated on Plate and Subsequently Encapsulated…………………………………………………………………………….7**
6. **Fraction Viable of Monocytes and Macrophages Differentiated in the Hydrogel…………………………………………………………………………….…...8**
7. **Flow Cytometry Data for CD68 Macrophage Marker ………………………………9**
8. **Non-Specific Binding of DBCO dye to Macrophages following SPAAC Click Reaction After Invasion……………………………………………………………………...……10**
9. **Application of DBCO-488 to Fixed Cells Test………………………………………...11**
10. **Application of DBCO-488 to Live Cells Test ………………………………….…..….12**
11. **Invasion of *E. coli* into Macrophages in 3D culture…………………………………..13**
12. **MS Spectra of Peptides………………………………………………………….……...14**
13. **NMR Spectra of PEG-8-Nb …………………………………………………………....16**
14. **References…………………………………………………………………………….…17**
15. **General Materials and Methods**

**Materials.**

All chemicals were purchased from Millipore-Sigma and ThermoFisher Scientific and used without further purification unless otherwise noted. Antibiotics were purchased from Gold Biotechnology. Deuterated NMR solvents were purchased from Cambridge Isotope Laboratories. *E. coli* *ΔMurQ-KU* strain and AzNAM probe were obtained from previous stocks in the Grimes Lab (1). THP-1, HEK2973T, and CCL151 cells were purchased from American Type Culture Collection (ATCC). Cell culture plates, tubes, and other consumables were purchased from Cell Treat and cell culture media was purchased from Corning.

**Instrumentation.**

NMR spectra were recorded on AV III 600 MHz spectrometers at the University of Delaware NMR Facility. Mass spectra (LRMS, ESI) were obtained using an ACQUITY UPLC H-Class/SQD2 at the University of Delaware Mass Spectrometry Facility. Peptides were synthesized using an automated peptide synthesizer (PS3, Protein Technologies and Liberty Blue; CEM, Matthews, NC) and were purified on reverse-phase high performance liquid chromatography (HPLC, XBridge BEH C18 OBD 5 μm column; Waters, Milford, MA). Rheometry was performed on AR-G2 rheometer with UV-visible light attachment (TA instruments) in tandem with an Omnicure Series 2000 light source (Excilitas) with a 365 nm bandpass filter and light guide (Exfo). Confocal microcopy images were taken on a Zeiss LSM800 instrument with Plan-Apochromat 63X/1.40 Oil DIC M27 objective. Super-resolution microscopy images were taken on Zeiss Elyra PS.1 microscope with Plan-Apochromat 63x/1.4 oil differential interference contrast (DIC) M27 objective. Live cell imaging was done on Andor Dragonfly 505 spinning disk confocal microscope with a Plan-Apochromat 63x/1.47 oil objective.

1. **Concentration and Reaction Time Course of SPAAC Reaction on *E. coli* Peptidoglycan**

**
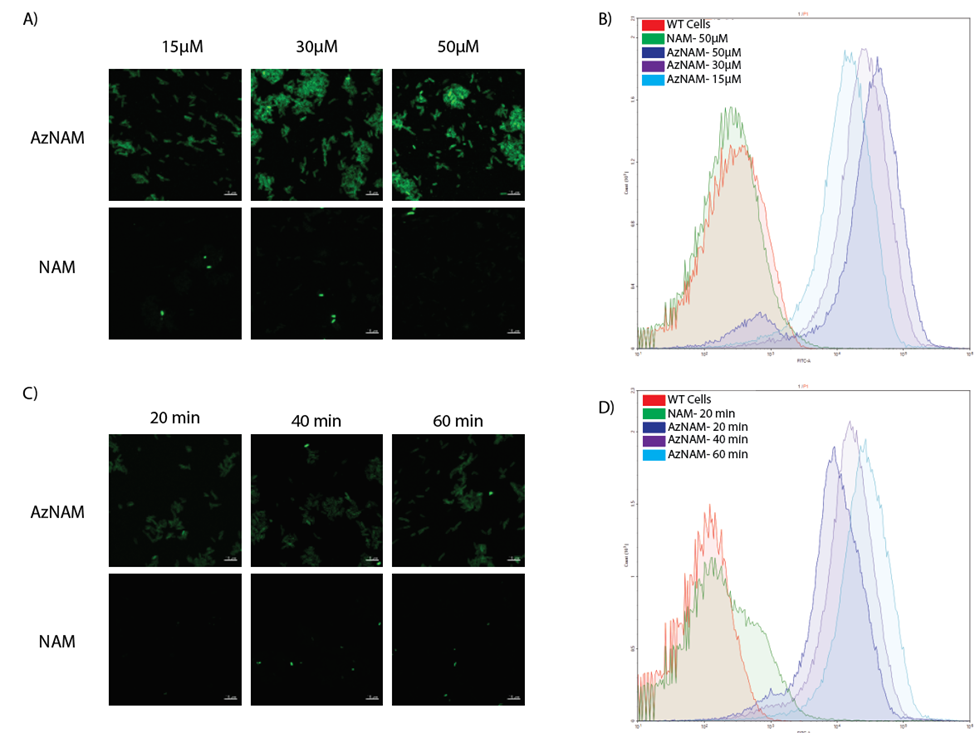
**

**SI Figure 1:** **A)** EQKU cells were remodeled with NAM or AzNAM, and then treated with various concentrations of DBCO probe for 40 min at 37°C. Images were taken on LSM800 confocal microscope and are representative of a minimum of three fields of view per sample and three biological replicates. **B)** Flow cytometry data of the remodeled and clicked EQKU samples shows that all concentrations result in a high percentage of labeled cells. However, at a concentration of 50μM DBCO probe, a secondary population cells hypothesized to be dead or dying cells appears. **C)** EQKU cells were remodeled with NAM or AzNAM, and then treated with 30 μM DBCO probe various lengths of time at 37°C. Images taken at 20 min appear to be less bright than longer incubation times. Images were taken on LSM800 confocal microscope and are representative of a minimum of three fields of view per sample and three biological replicates. **D)** Flow cytometry data of the remodeled and clicked EQKU samples show that all of the incubation periods with the DBCO result in a high percentage of labeled cells.

1. **Growth curve analysis of SPAAC Reaction of EQKU**

**
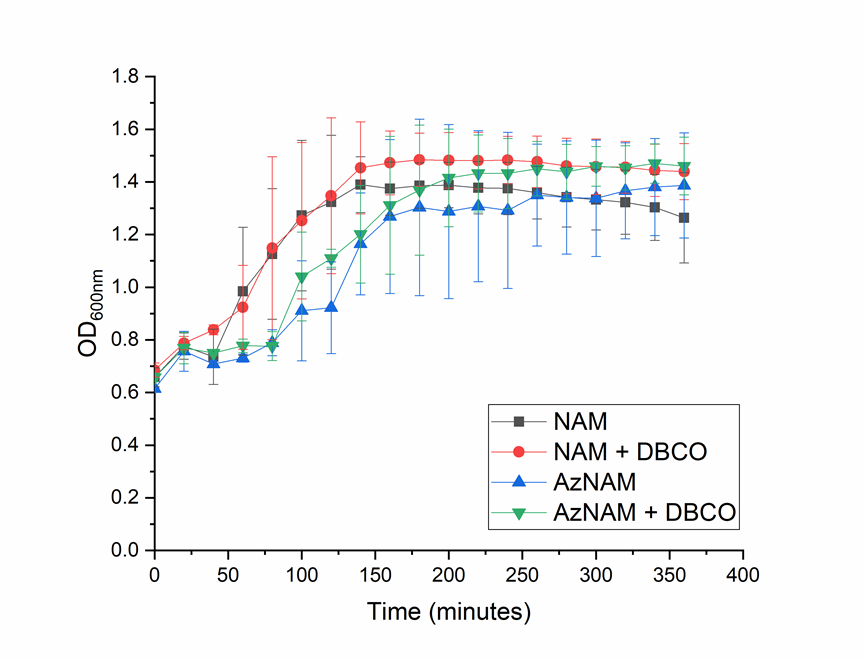
**

**SI Figure 2:** EQKU cells were remodeled with NAM or AzNAM probe. Cells were added to a 96-well plate and specific conditions (‘+ DBCO’) were given the DBCO probe, which would react with azide during a SPAAC reaction. The AzNAM + DBCO sample did not exhibit any changes in cell growth upon the reaction taking place on the bacterial PG in comparison to the controls (NAM, NAM + DBCO, and AzNAM), which either did not contain the DBCO or lacked the azide handle to react with the DBCO.

1. **Growth Curve Analysis of SPAAC Reaction of *P. aeruginosa***

**
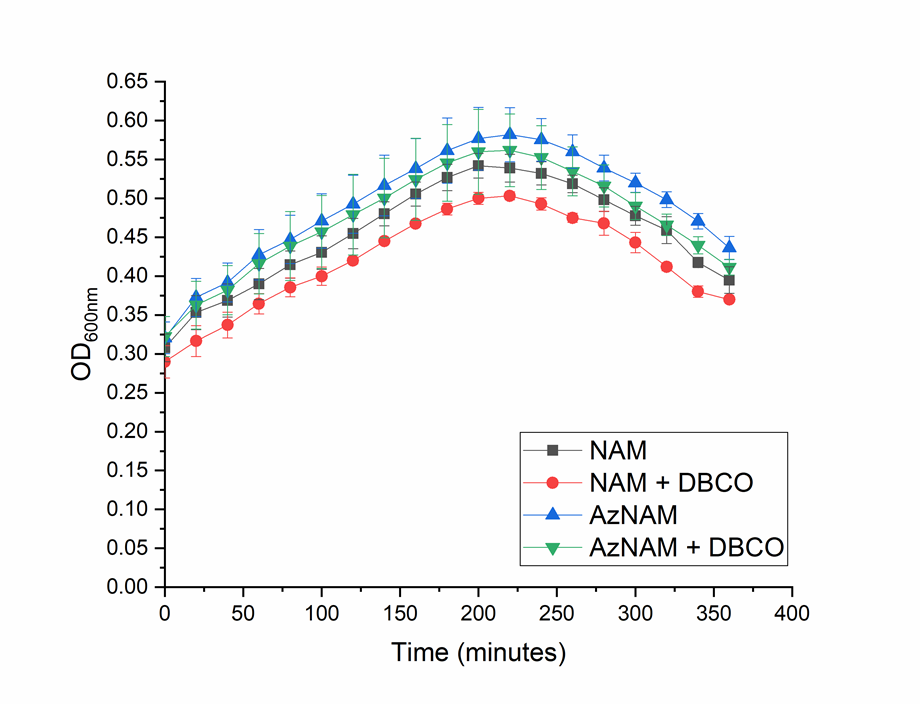
**

**SI Figure 3:** *P. aeruginosa* cells were remodeled with NAM or AzNAM probe. Cells were added to a 96-well plate and specific conditions (‘+ DBCO’) were given the DBCO probe, which would react with azide during a SPAAC reaction. The AzNAM + DBCO sample did not exhibit any changes in cell growth upon the reaction taking place on the bacterial PG in comparison to the controls (NAM, NAM + DBCO, and AzNAM), which either did not contain the DBCO or lack the azide handle to react with the DBCO.

1. **Live/Dead of Macrophages Differentiated on Plate and Subsequently Encapsulated**

**
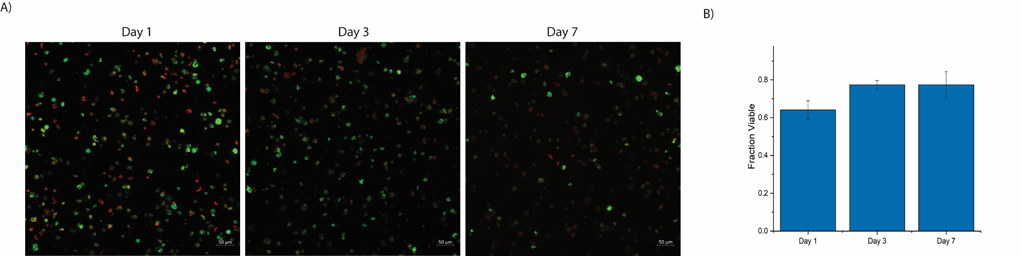
**

**SI Figure 4:** A) Representative confocal microscope images (maximum intensity projections) of live/dead cytotoxicity assay results from 3D culture of THP-1 cells differentiated with PMA into macrophages on tissue culture polystyrene and then encapsulated within hydrogels. Scale bars = 50μm B) Quantification of fraction viable over time (replicates associated with representative images shown in A) demonstrated viability less than 80%, which was viability observed when THP-1 monocytes were encapsulated in the hydrogels and subsequently differentiated (see SI Figure 2).

1. **Fraction Viable of Monocytes and Macrophages Differentiated in the Hydrogel**

**
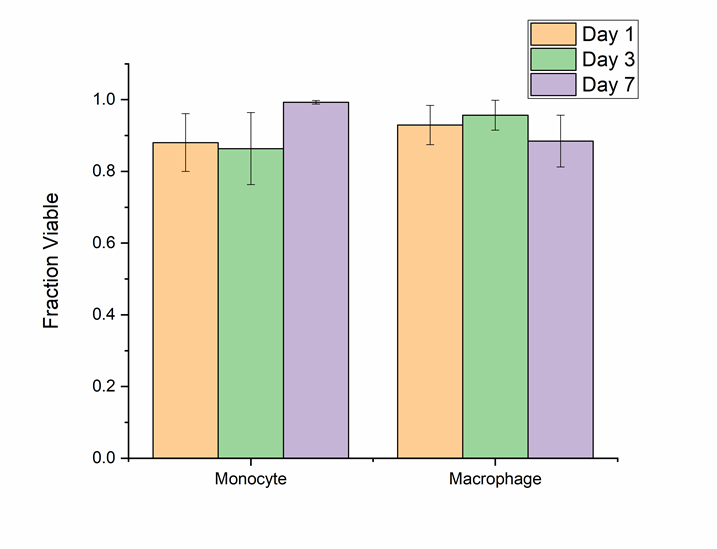
**

**SI Figure 5:** Quantification of fraction viable THP-1 monocyte cells and THP-1 macrophage cells in 3D culture, which were encapsulated as monocytes and subsequently differentiated with TPA during 3D culture. These analyses demonstrated high viability for both monocytes and macrophages in 3D culture (above ~ 85% and 90%, respectively) within the hydrogel-based synthetic extracellular matrices.

1. **Flow Cytometry Data for CD68 Macrophage Marker in 2D and 3D Culture**

**
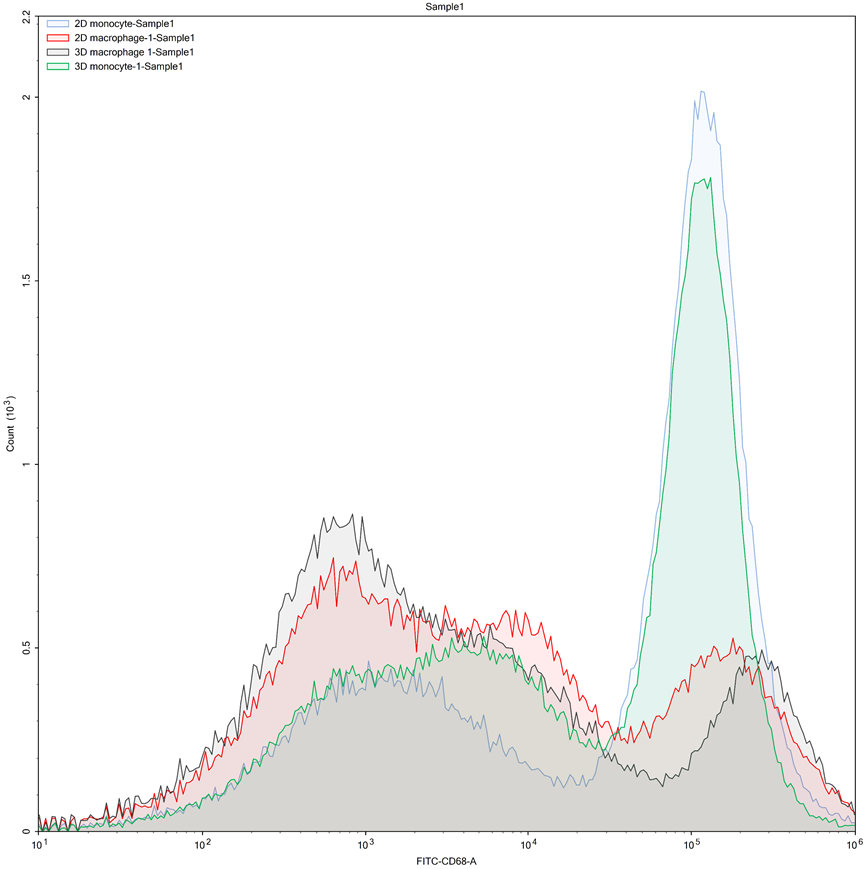
**

**SI Figure 6:** Flow cytometry data of macrophages and monocytes in 2D and 3D culture stained for extracellular macrophage marker CD68. 2D (blue) and 3D culture monocyte (green) profiles match each other, and 2D (red) and 3D (black) culture macrophage profiles match each other, showing differentiation was achieved in 3D culture within hydrogels. This data supports the data shown in the main text for macrophage marker CD11b (Figure 3).

1. **Non-Specific Binding of DBCO Dye to Macrophages Following SPAAC Click Reaction After Invasion**


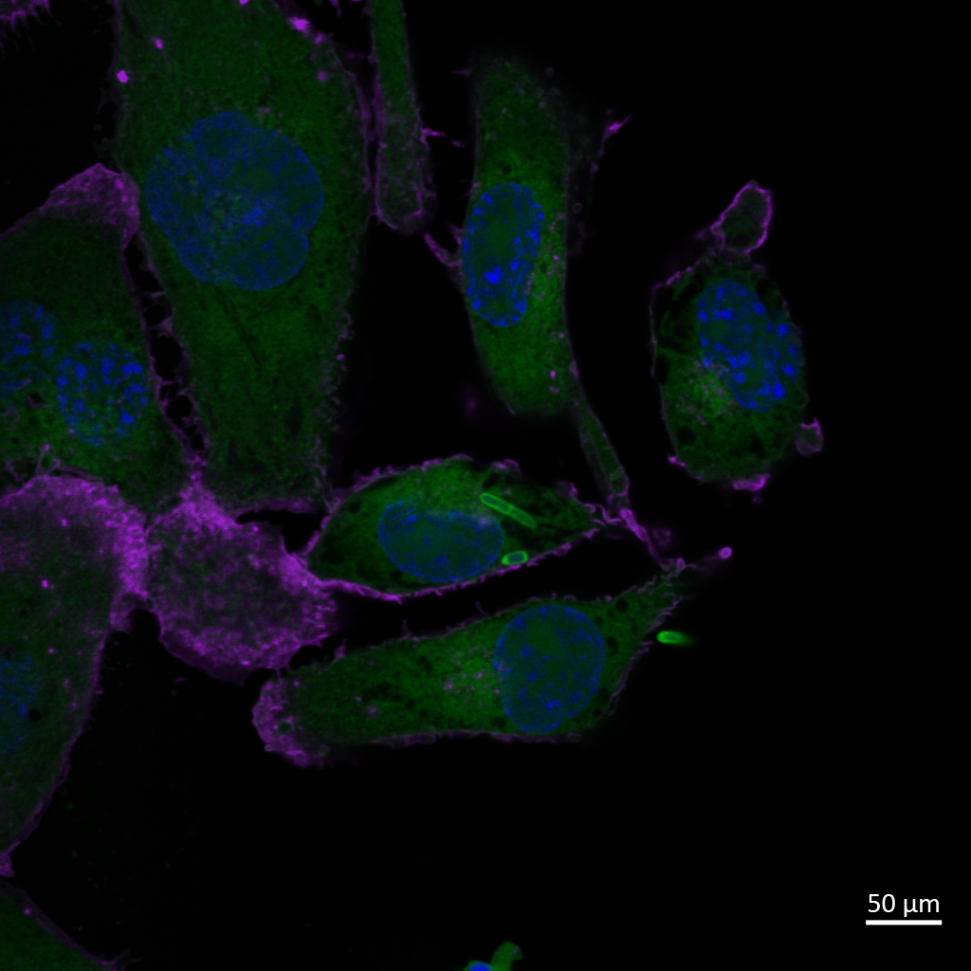


**SI Figure 7:** E. *coli ΔMurQ KU* cells are remodeled by incorporating the azido bacterial cell wall precursor in the cell wall with recycling enzymes AmgK and MurU. Bacteria were invaded into THP-1 macrophage cells and fixed prior to the SPAAC click labeling (bacteria, green) and subsequent staining with Phalloidin-TRITC (F-actin, purple) and Hoechst (nucleus, blue). Macrophages exhibit non-specific binding of the DBCO fluorophore through this labeling methodology. Images were taken on a Zeiss LSM800 Confocal Microscope. Images are representative of a minimum of three ﬁelds viewed per replicate with at least two technical replicates, and experiments were conducted in at least three biological replicates. Scale bars = 50 μm.

1. **Application of DBCO-488 to Fixed Cells Test**

**
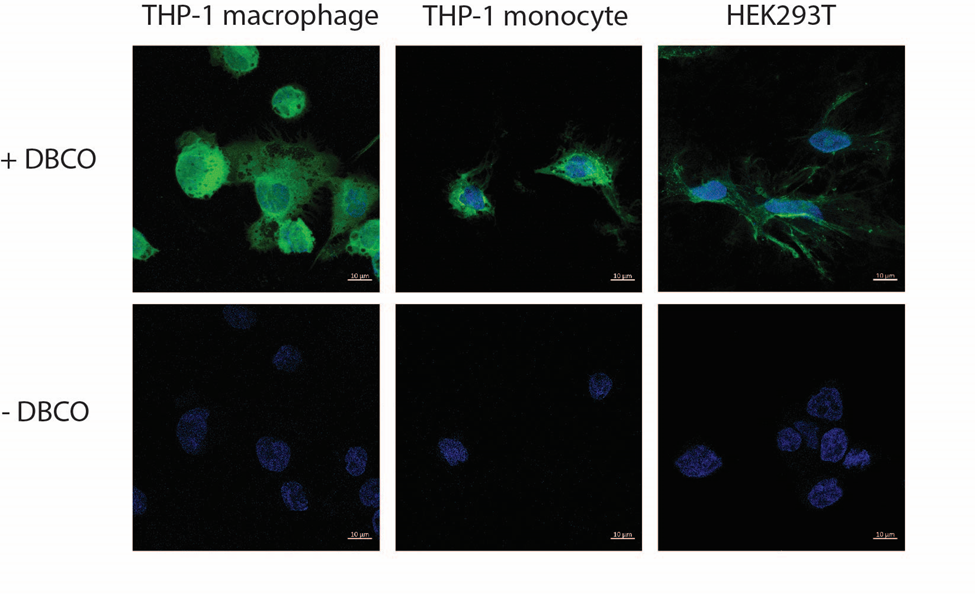
**

**SI Figure 8:** Cells were fixed and some were treated with 30μM DBCO-488. All cell types treated with DBCO exhibited non-specific binding of the DBCO dye as compared to the control. (Blue = nucleus; green = DBCO-488) Scale bars = 10μM

1. **Application of DBCO-488 to Live Cells Test**

**
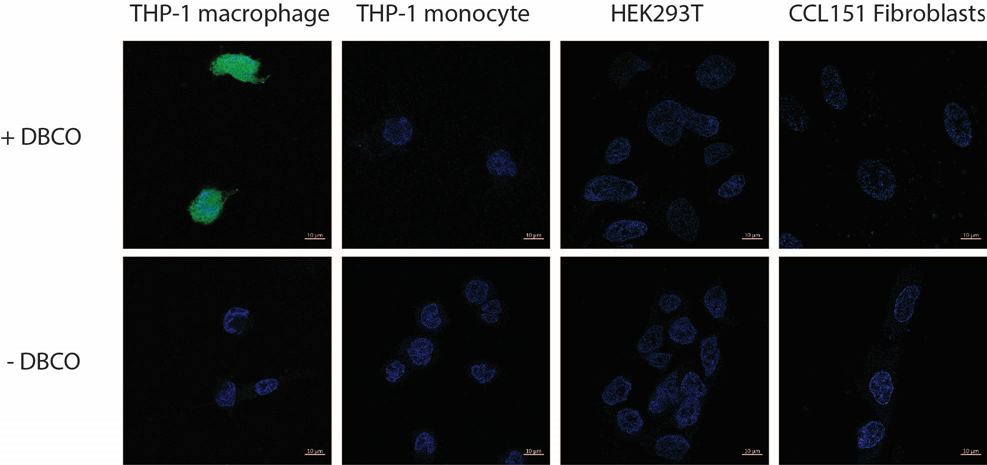
**

**SI Figure 9:** Live cells were treated with 30μM DBCO-488 or water. Cells were then washed, fixed and mounted with DAPI mounting media. Only THP-1 macrophages treated with DBCO-488 exhibited non-specific binding of the DBCO dye as compared to the controls. (Blue = nucleus; green = DBCO-488) Scale bars = 10μM

1. **Invasion of *E. coli* into Macrophages in 3D culture**


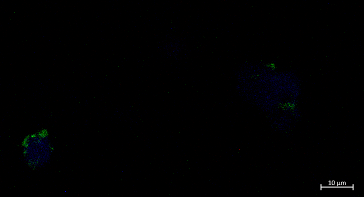


**SI Figure 10:** Confocal microscopy z-stack projections of *E. coli ΔMurQ KU* cells invaded into THP-1 macrophages cells encapsulated in hydrogel-based synthetic matrix for 3D culture. All cells were fixed and Alk488 was clicked on to the remodeled bacteria (green). Nuclei were labeled with 4,6-diamidino-2-phenylindole (blue) (scale bar 10 µM). Image is representative of a minimum of three ﬁelds viewed per replicate with at least two technical replicates, and invasion experiments were conducted in at least three biological replicates.

1. **MS of Peptides**

**
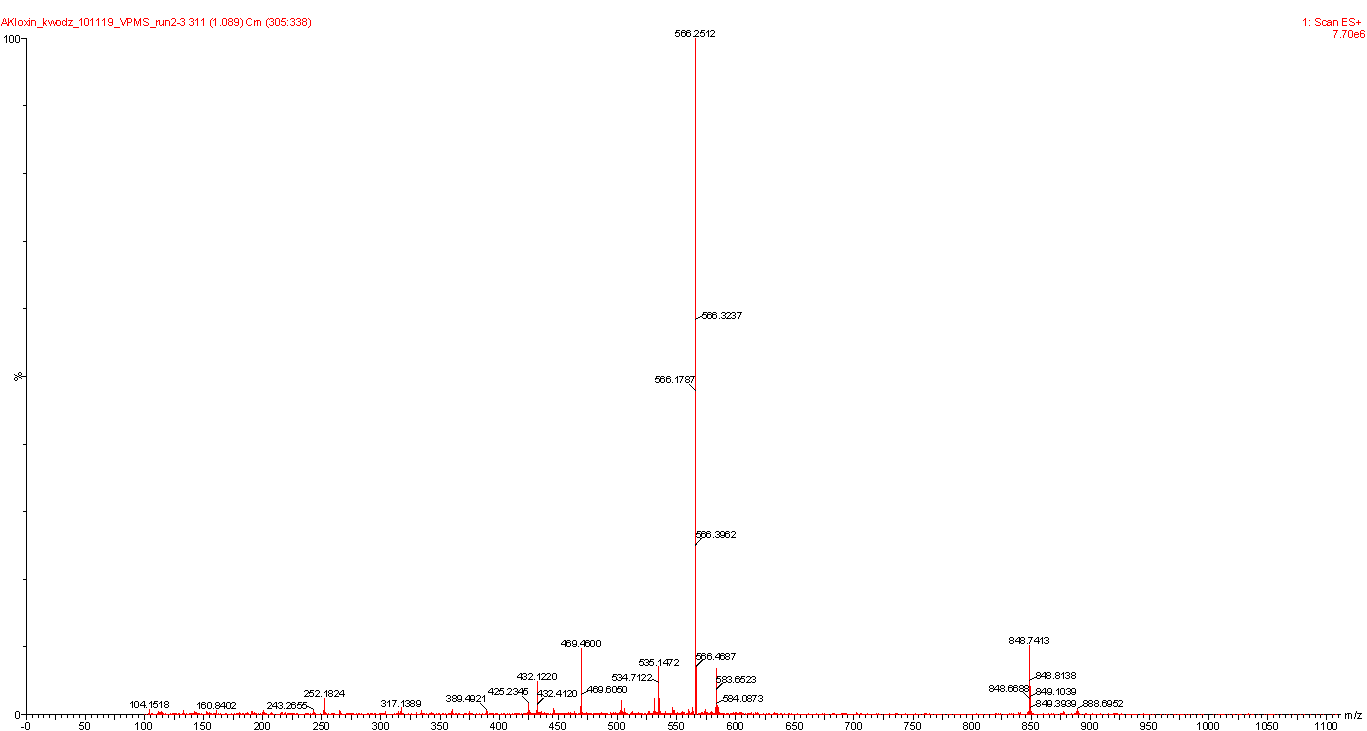
**

**SI Figure 11:** Mass spectrometry of difunctional linker peptide. Successful synthesis of crosslinking peptide, CGRDVPMSMRGGDRCG-amide, was confirmed by SQD2 mass spectrometry. Expected molecular weight of 1696 g/mol. [M + 2H]^+^ = 848 g/mol [M + 3H]^+^ = 566 g/mol.


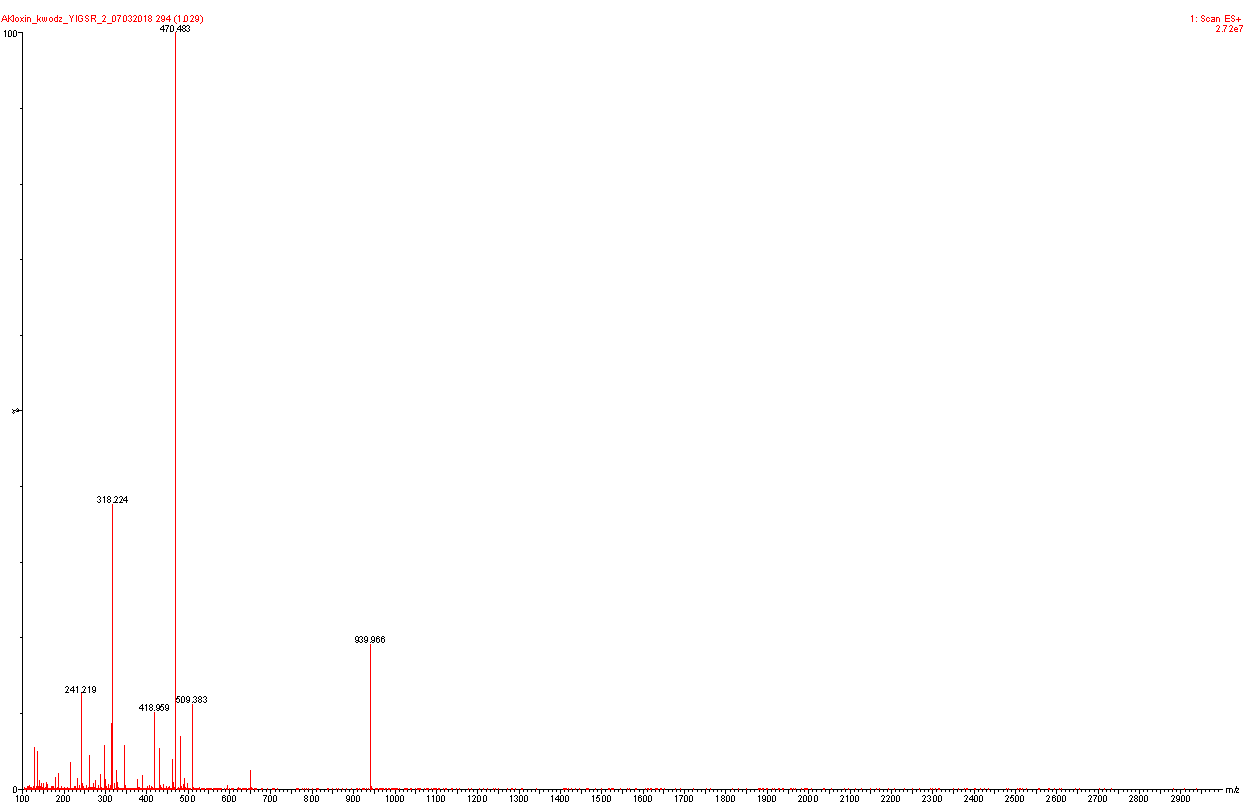


**SI Figure 12:** Mass spectrometry of monofunctional pendant peptide. Successful synthesis of pendant peptide, CGKGYIGSR-amide, was confirmed by SQD2 mass spectrometry. Expected molecular weight of 939 g/mol. [M + H]^+^= 940 g/mol. [M + 2H]^+^= 470 g/mol.

1. **NMR Spectra of PEG-8-Nb**


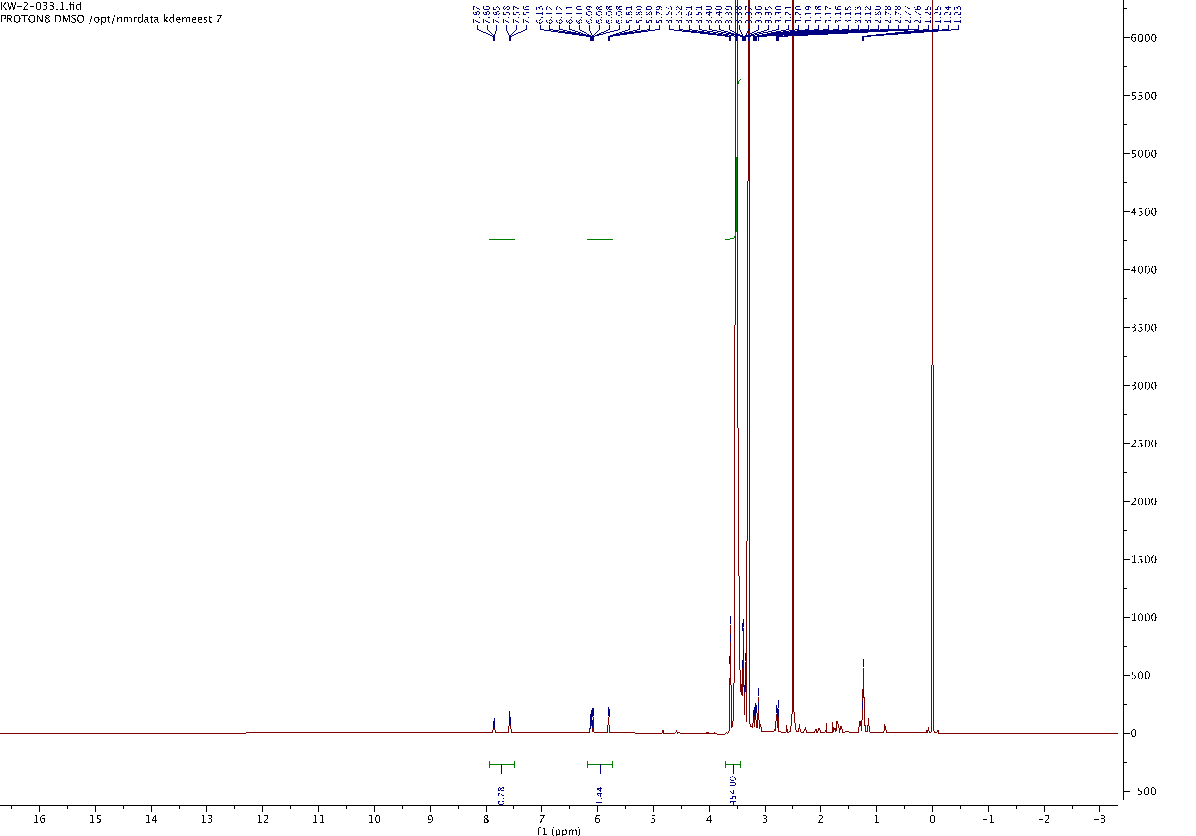


**SI Figure 13:** Representative ^1^H NMR of PEG-8-Nb. The functionality is based on the number of protons corresponding to norbornene normalized to the PEG backbone. With expected integration for norbornene protons (2H, 6.20 to 5.86 ppm) and the PEG backbone protons (454 H, 3.65 to 3.40 ppm), the calculated norbornene functionality was 1.44 divided by 2, which equals 72% functionality for this batch, where ~ 75% functionality was observed on average between batches.

1. **References**

1. Liang H, DeMeester KE, Hou CW, Parent MA, Caplan JL, Grimes CL. Metabolic labelling of the carbohydrate core in bacterial peptidoglycan and its applications. Nat Commun. 2017;8:15015.
